# Supplementary figures and images for: Quantitative multi-pathway assessment of exposure to Escherichia coli for infants in Rural Ethiopia
Source: PLoS Negl Trop Dis. 2025 Jun 9;19(6):e0013154. doi: 10.1371/journal.pntd.0013154 (PMC12176293; doi:10.1371/journal.pntd.0013154)

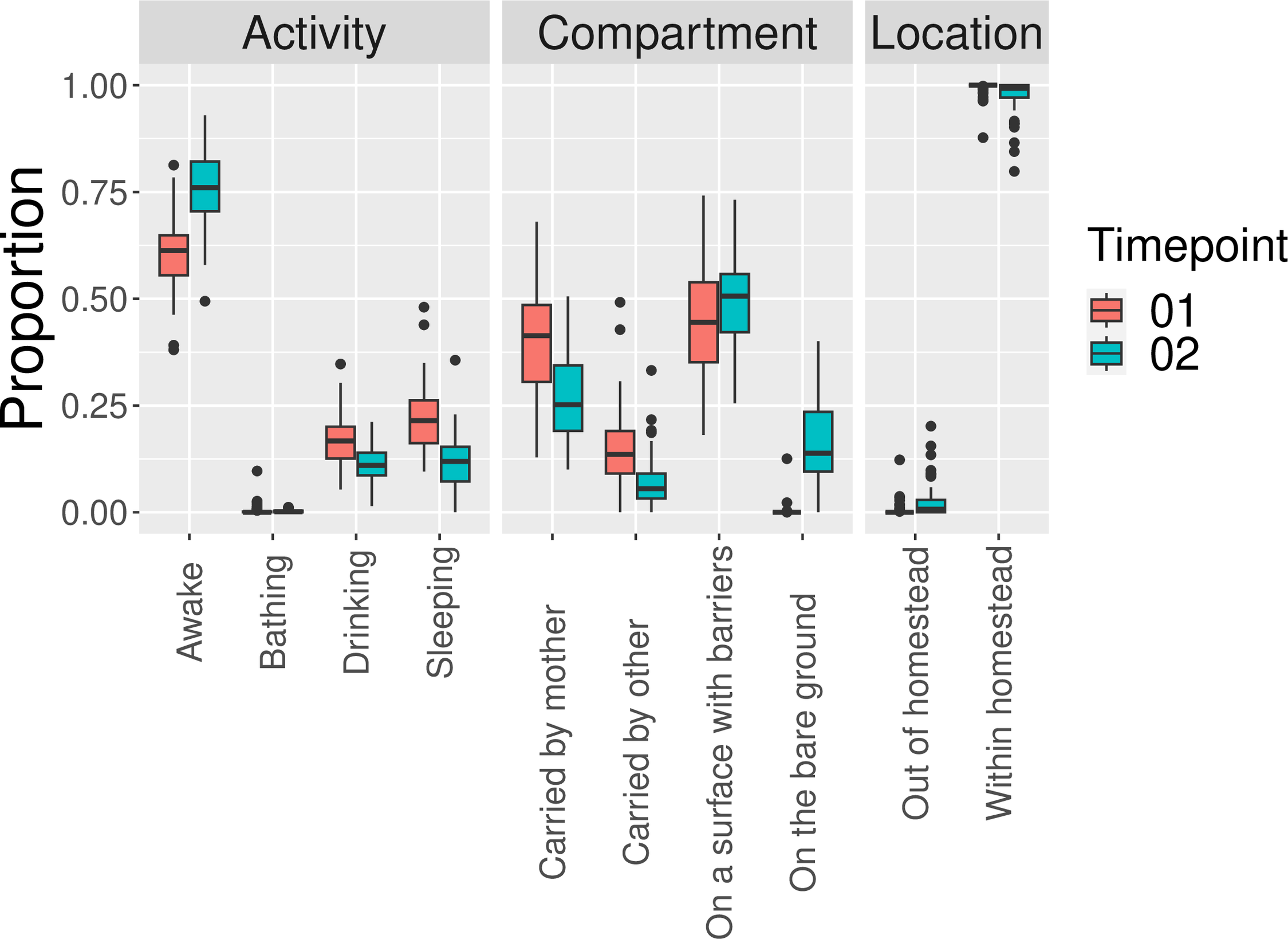

Supplement: S1 Fig — Boxplots show distributions of the proportions of time spent on specific activities, compartments, or locations among infants. The central line in the box indicates the median value, while the box limits indicate the first and third quartile. The points outside of the box indicate outliers (TIF) [file pntd.0013154.s001.tif]

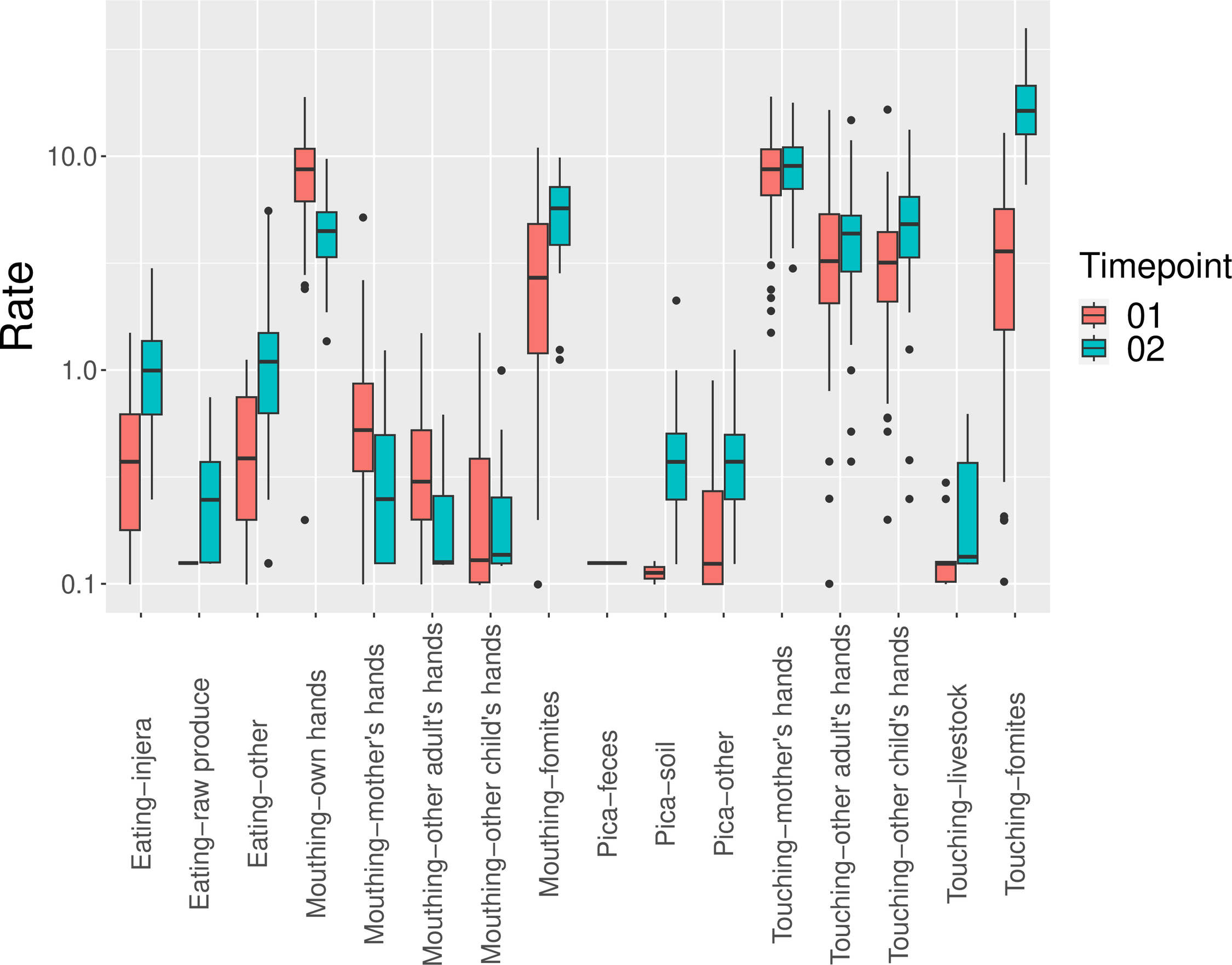

Supplement: S2 Fig — Boxplots show distributions of the rates of frequency-based behaviors among infants. The central line in the box indicates the median value, while the box limits indicate the first and third quartile. The points outside of the box indicate outliers. (TIF) [file pntd.0013154.s002.tif]

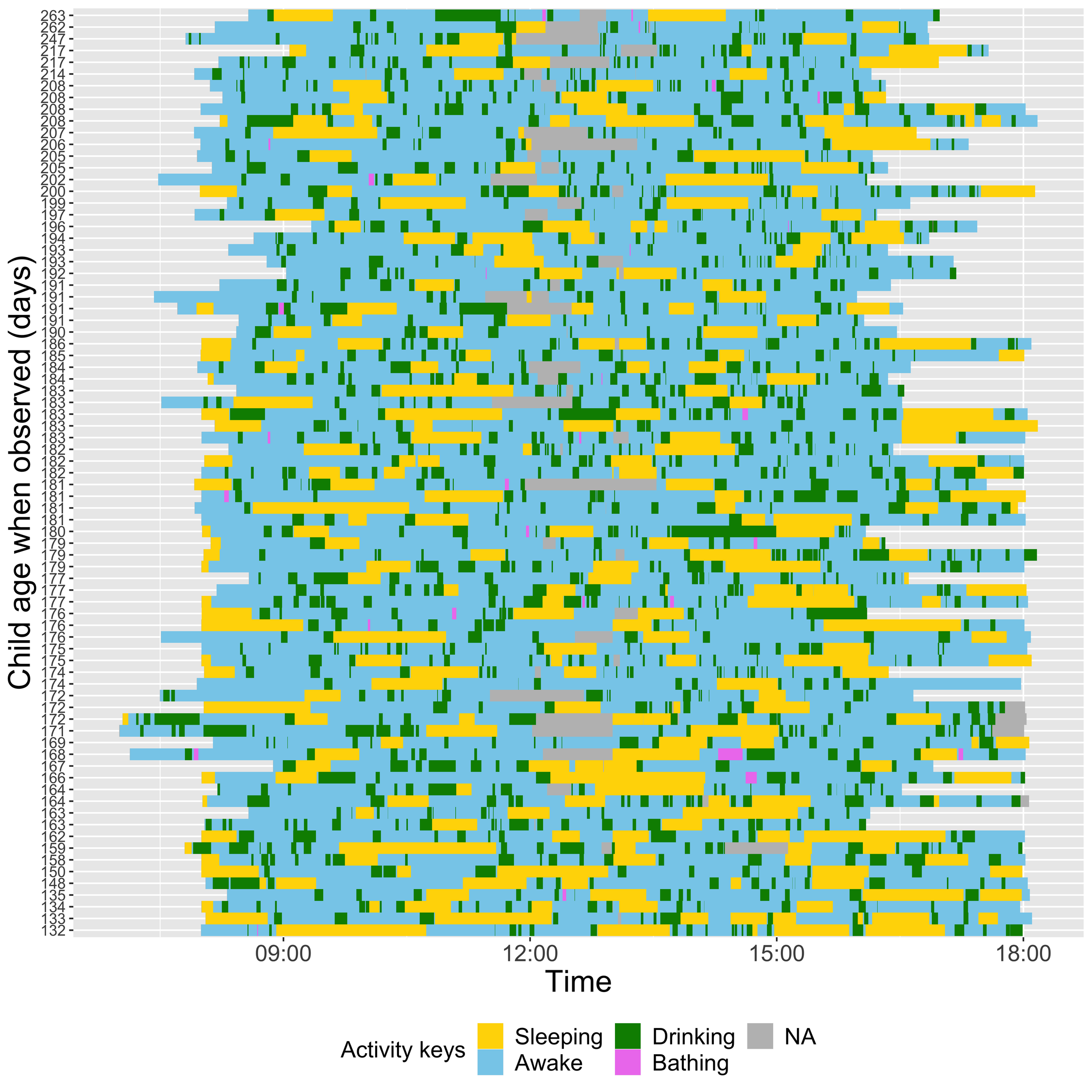

Supplement: S3 Fig — The morning and afternoon sessions were combined for each infant. The x-axis shows the time and the y-axis shows the age of infants, which is sorted in ascending order (from bottom to top). “NA” represents the time period not observed. (TIF) [file pntd.0013154.s003.tif]

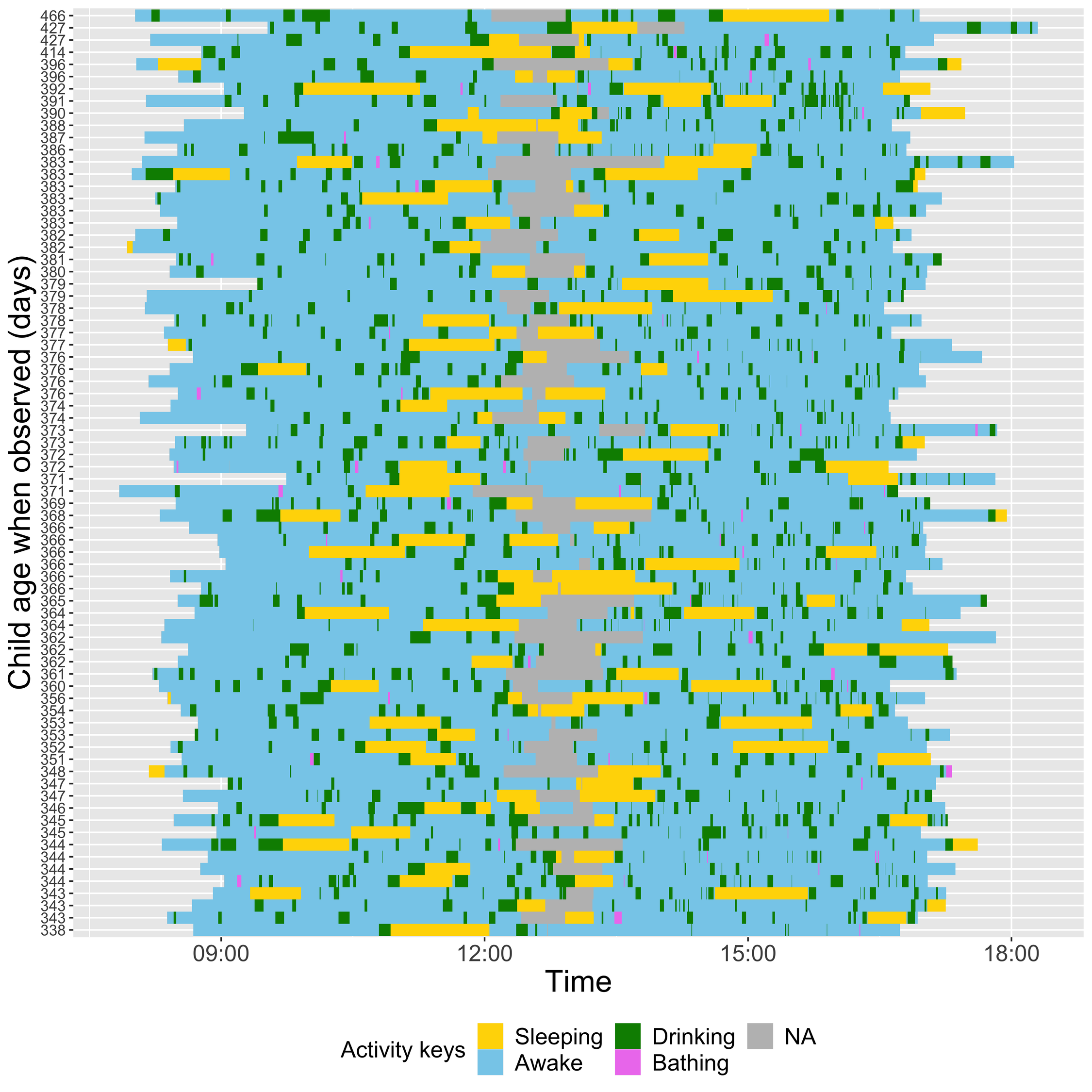

Supplement: S4 Fig — The morning and afternoon sessions were combined for each infant. The x-axis shows the time and the y-axis shows the age of infants, which is sorted in ascending order (from bottom to top). “NA” represents the time period not observed. (TIF) [file pntd.0013154.s004.tif]

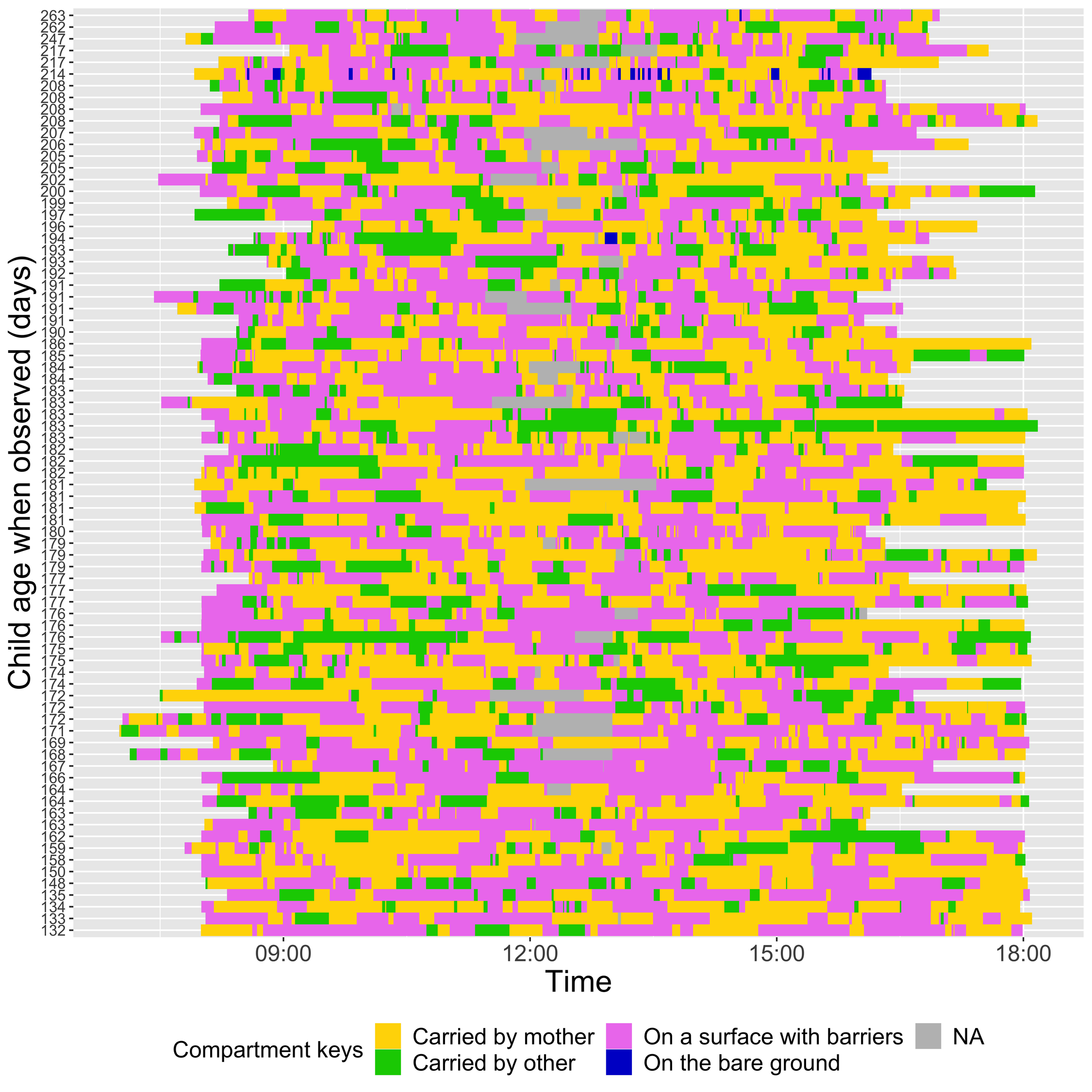

Supplement: S5 Fig — The morning and afternoon sessions were combined for each infant. The x-axis shows the time and the y-axis shows the age of infants, which is sorted in ascending order (from bottom to top). “NA” represents the time period not observed. (TIF) [file pntd.0013154.s005.tif]

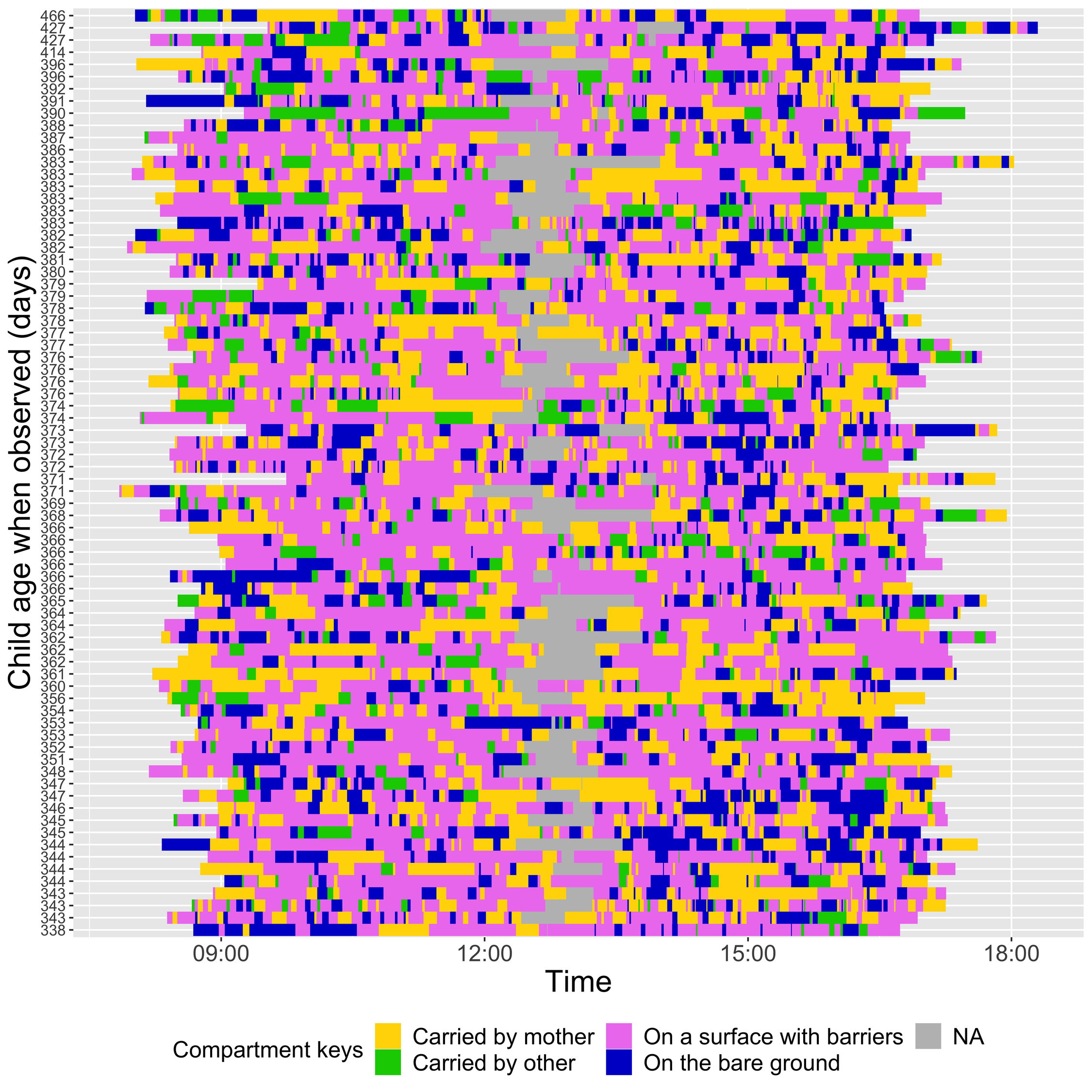

Supplement: S6 Fig — The morning and afternoon sessions were combined for each infant. The x-axis shows the time and the y-axis shows the age of infants, which is sorted in ascending order (from bottom to top). “NA” represents the time period not observed. (TIF) [file pntd.0013154.s006.tif]

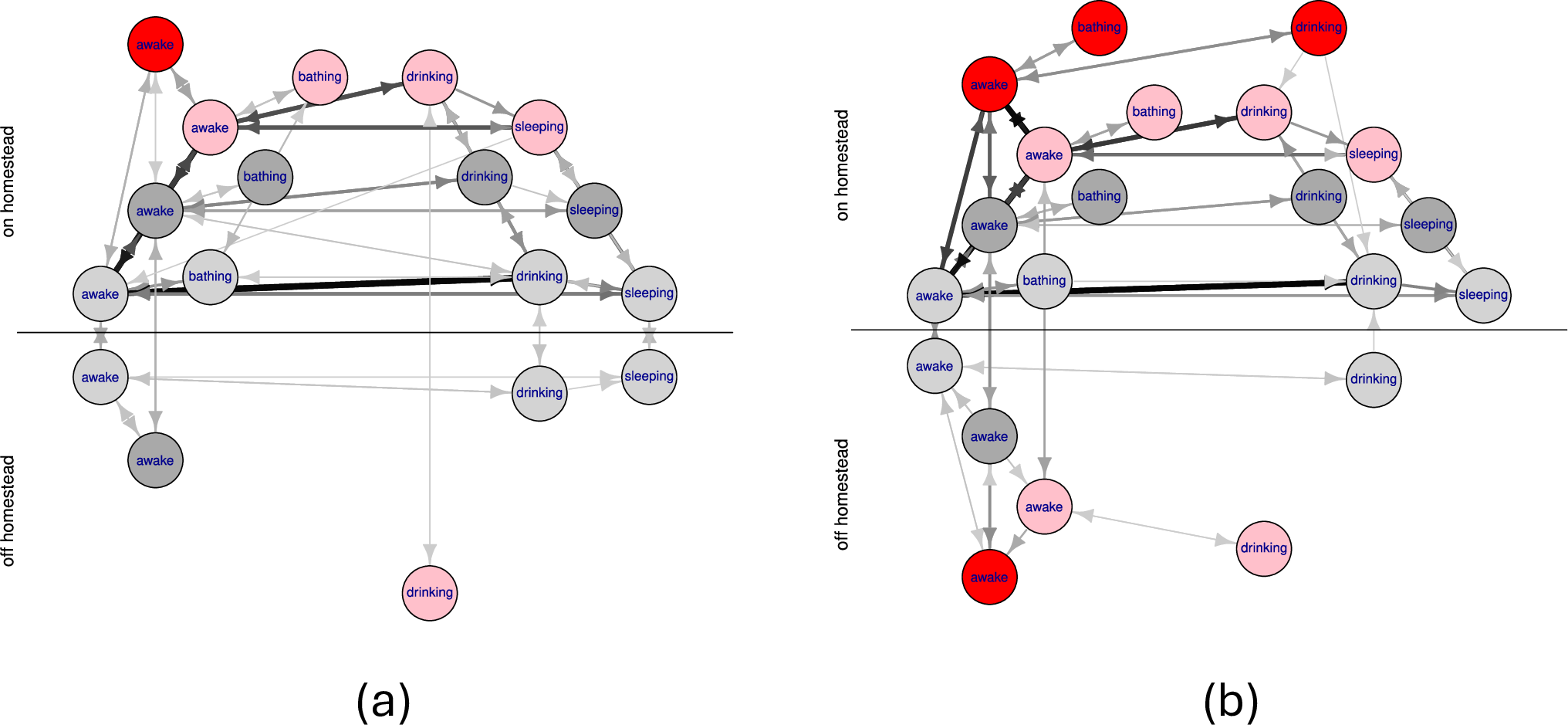

Supplement: S7 Fig — (a) Observed behavior transition network at Timepoint 1 (6050 transitions); (b) observed behavior transition network at Timepoint 2 (6568 transitions). The text inside the node shows the activity. The color of the node represents the compartment: light gray is Carried by Mother; dark gray is Carried by Others; pink is Down on a Surface with Barriers; red is Down on the Bare Ground. For the location, the nodes above the horizontal line are within homestead, while those below the horizontal line are out of homestead. Arrows indicate transitions between states (i.e., combinations of activity, compartment, and location), and strengths (numbers of times the transition was observed) indicated by arrow width and shade (darker arrows indicate higher frequency). (TIF) [file pntd.0013154.s007.tif]

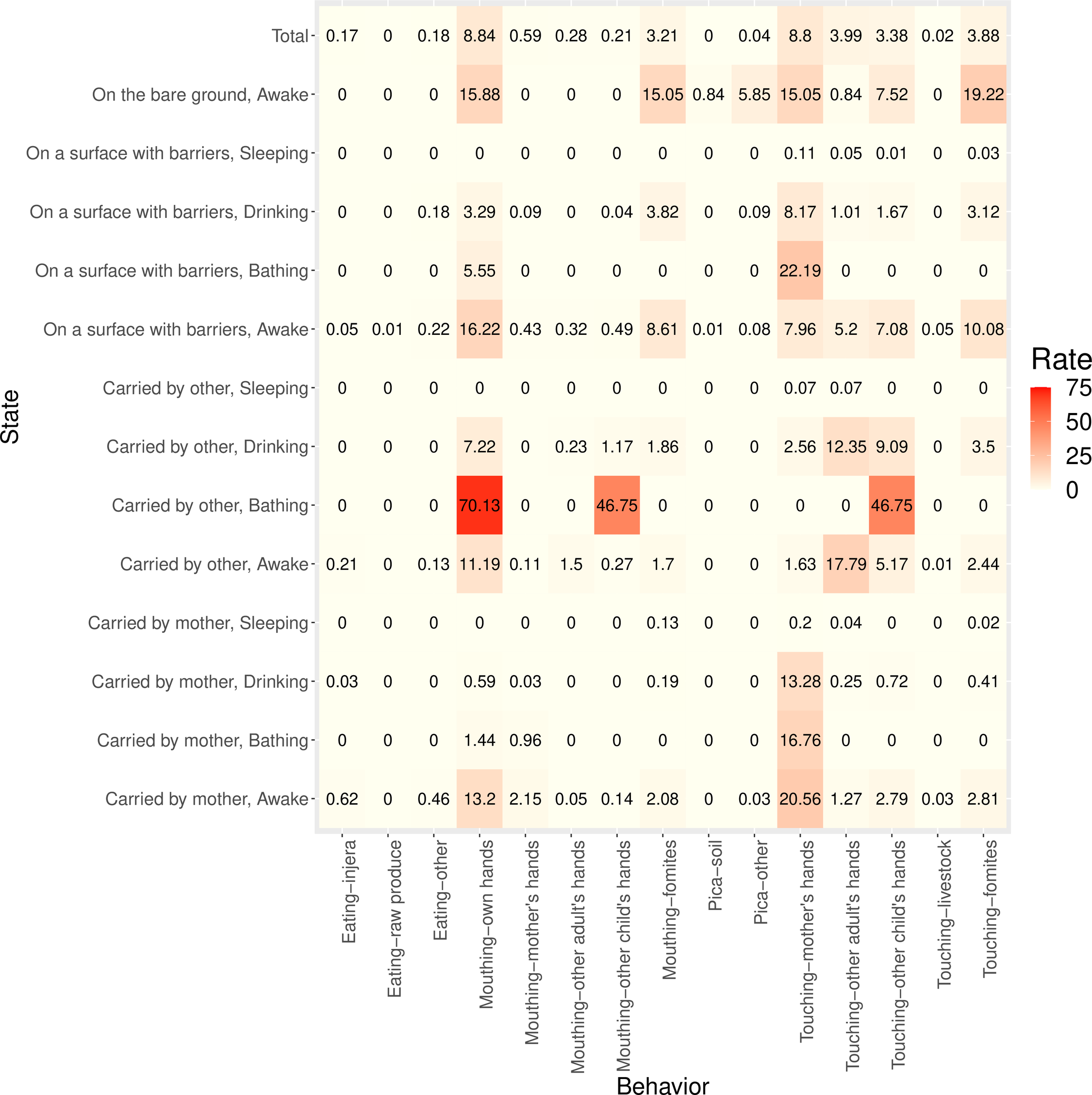

Supplement: S8 Fig — The color represents the rate: darker colors show higher rates. (TIF) [file pntd.0013154.s008.tif]

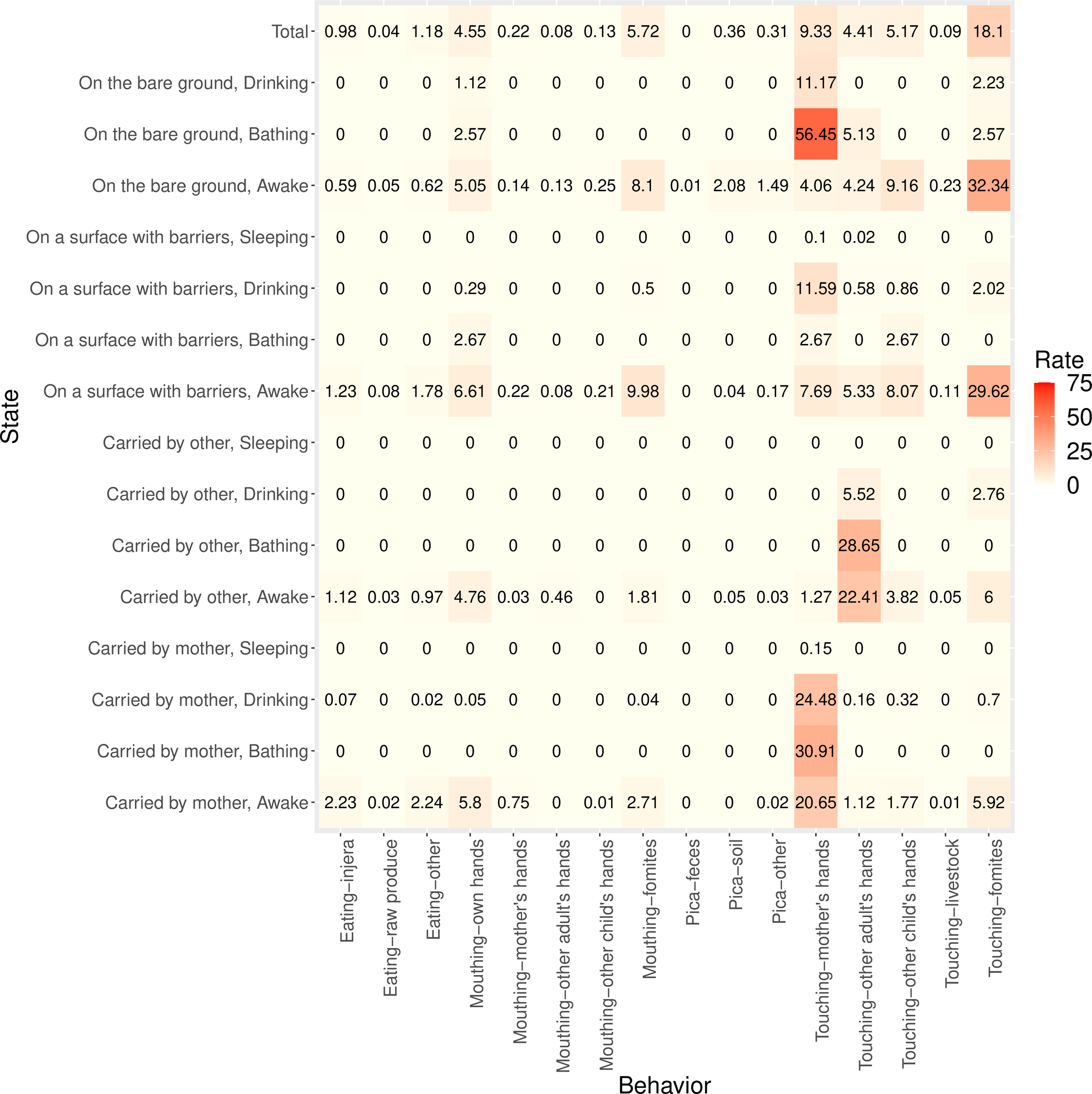

Supplement: S9 Fig — The color represents the rate: darker colors show higher rates. (TIF) [file pntd.0013154.s009.tif]

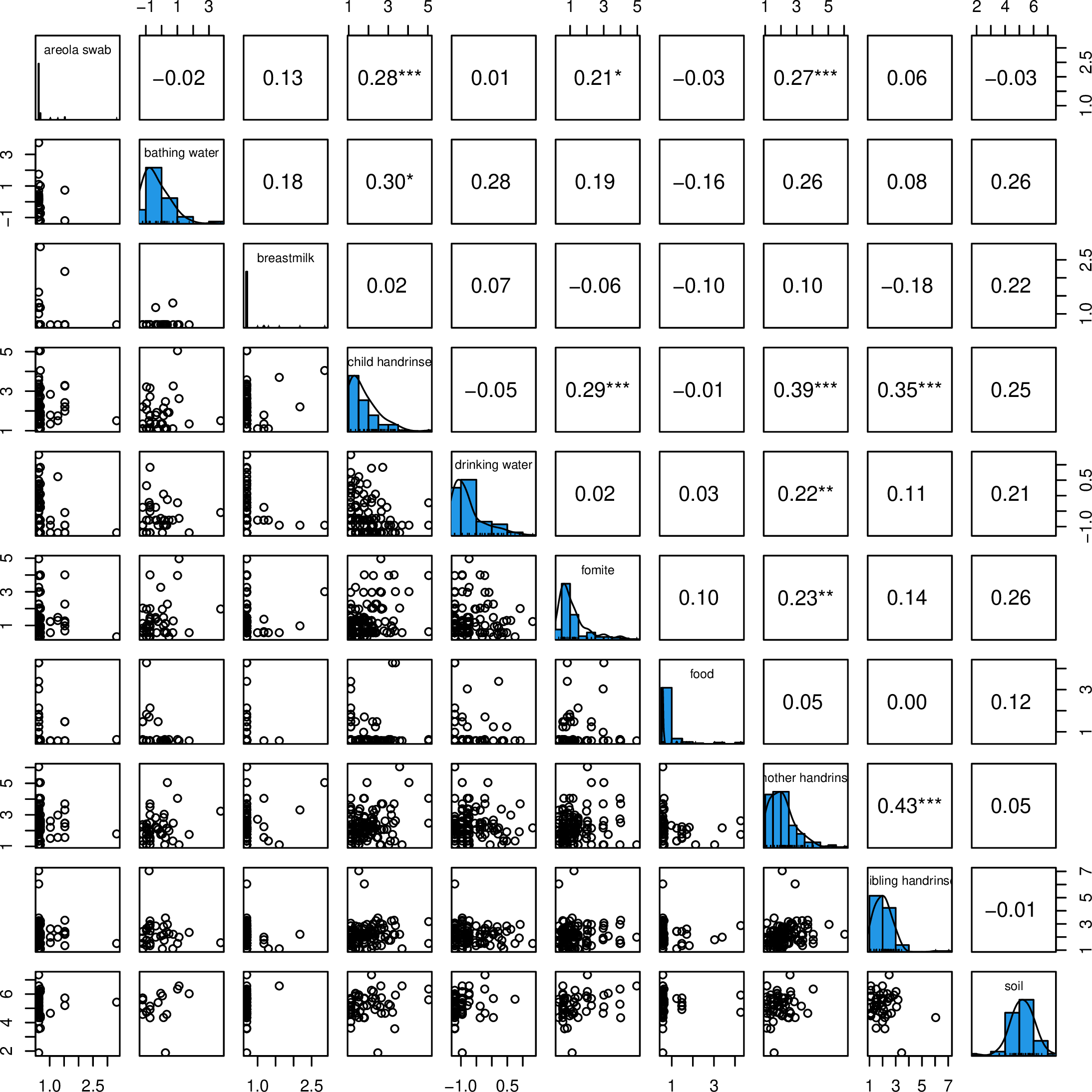

Supplement: S10 Fig — In the correlation matrix, plots in the bottom left half show the scatterplots of log10 scale E. coli concentration levels for pairs of sample types. Plots on the diagonal line show the histogram of log10 scale E. coli concentration levels by sample type. Plots on the top right half show the Spearman correlation coefficients of log10 scale E. coli concentration levels between different sample types. The stars in the figure define the level of significance. * = 0.05, ** = 0.01, *** = 0.001. (TIF) [file pntd.0013154.s010.tif]

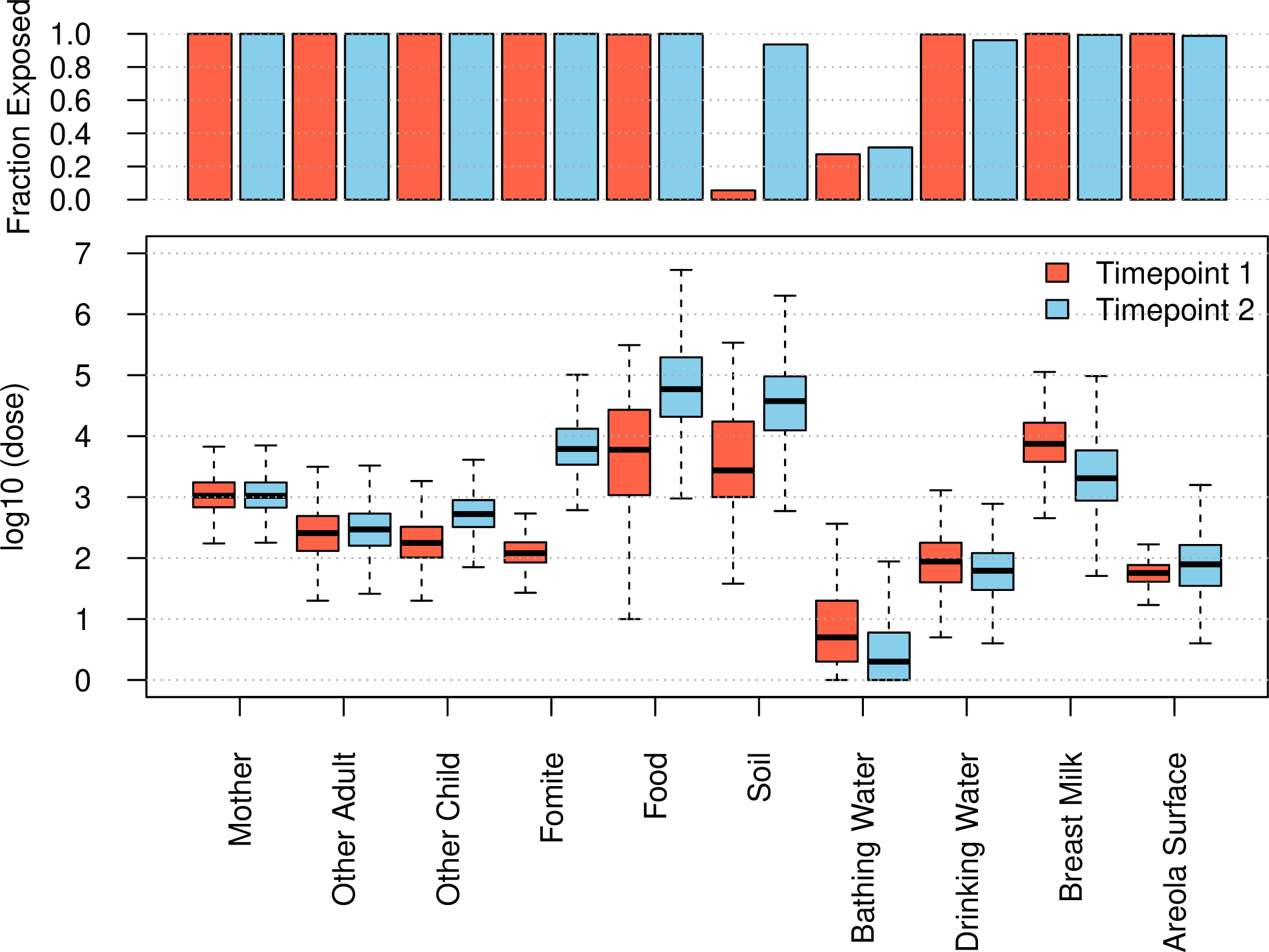

Supplement: S11 Fig — The bar charts show the fraction of simulated days that children are exposed, and boxplots show the estimated daily dose of E. coli (log10 CFU/day). (TIF) [file pntd.0013154.s011.tif]
