# Supplementary material for: Quantitative multi-pathway assessment of exposure to Escherichia coli for infants in Rural Ethiopia
Source: PLoS Negl Trop Dis. 2025 Jun 9;19(6):e0013154. doi: 10.1371/journal.pntd.0013154 (PMC12176293; doi:10.1371/journal.pntd.0013154)
Supplement: S1 Appendix — (PDF) [file pntd.0013154.s012.pdf]

Quantitative Multi-pathway Assessment of Exposure to *Escherichia coli* for Infants in Rural Ethiopia

Yuke Wang[1*], Yang Yang[2], Crystal M. Slanzi[3], Xiaolong Li[4,5], Amanda Ojeda[6], Fevi Paro[4], Loïc Deblais[7,8], Habib Yakubu[1], Bahar Mummed Hassen[9], Halengo Game[10], Kedir Teji Roba[10,11], Elizabeth Schieber[12], Abdulmuen Mohammed Ibrahim[10], Jeylan Wolyie[13], Jemal Yusuf Hassen[10], Gireesh Rajashekara[7,8,14], Sarah L. McKune[4], Arie H. Havelaar[4], Christine L. Moe[1], Song Liang[15]

1 Hubert Department of Global Health, Rollins School of Public Health, Emory University, Atlanta, Georgia, United States of America
2 Department of Statistics, Franklin College of Arts and Sciences, University of Georgia, Athens, Georgia, United States of America
3 Department of Psychology, California State University, Los Angeles, Los Angeles, California, United States of America
4 Emerging Pathogens Institute, University of Florida, Gainesville, Florida, United States of America
5 Department of Environmental and Global Health, College of Public Health and Health Professions, University of Florida, Gainesville, Florida, United States of America
6 Department of Microbiology and Cell Science, University of Florida, Gainesville, Florida, United States of America
7 Department of Animal Sciences, CFAES Wooster, The Ohio State University, Wooster, Ohio, United States of America
8 Global One Health initiative, The Ohio State University, Addis Ababa, Ethiopia
9 College of Veterinary Medicine, Haramaya University, Dire Dawa, Ethiopia
10 College of Health and Medical Sciences, Haramaya University, Dire Dawa, Ethiopia
11 Department of Biobehavioral Health, Pennsylvania State University, University Park, Pennsylvania, United States of America
12 dfusion Inc., Scotts Valley, California, United States of America
13 College of Social Sciences and Humanities, Haramaya University, Dire Dawa, Ethiopia
14 Department of Pathobiology, College of Veterinary Medicine, University of Illinois at Urbana-Champaign, Urbana, Illinois, United States of America
15 Department of Environmental Health Sciences, School of Public Health and Health Sciences, University of Massachusetts, Amherst, Massachusetts, United States of America

* yuke.wang@emory.edu

Appendix

Exposure Model and Parameters

Our exposure model includes oral ingestion through direct pathways and indirect pathways. Direct pathway is defined as *source* to *mouth*, such as eating contaminated food and drinking contaminated water. Indirect pathway is defined as *source* to *vehicle* to *mouth*, such as touching contaminated surfaces and then mouthing the hands.

Attachment and Detachment

In this model, hands play a critical role in transferring microbes from environmental and social *sources* to ingestion. Hand contacting a surface leads to a two-way microbe exchange: whenever a hand touches a contaminated surface, any microbe on the contacted surface may be transferred to the hand. At the same time, some of the previously attached microbes on the hands can be transferred to the contacted surface. In these two cases, given the probability of transfer is fixed, the number of microbes transferred from the hand to the surface (or from the surface to the hand) is a binomial sample of the total number that was present on the contact hand (or surface) area.

$$\begin{array}{lll} \text{Detachment} & N \to N - n & \binom{N}{n} p^n (1-p)^{N-n} \\ \text{Attachment} & N \to N + m & \binom{M}{m} q^m (1-q)^{M-m} \end{array}$$

where N is the number of microbes on the hand, n and m are the number of microbes detached and attached, respectively. And q and p are the attachment and detachment coefficients. The attachment and detachment of microbes happen simultaneously, so that the probability that a number $k = N - n + m$ remains on the hands is

$$P(k) = \sum_{n=0}^{N} \binom{N}{n} p^n (1-p)^{N-n} \binom{M}{n-N+k} q^{n-N+k} (1-q)^{M-n+N-k} \tag{1}$$

When repeatedly touching the same surface, the expected numbers of microbes may be easily calculated using

$$\begin{pmatrix} S_{n+1} \\ H_{n+1} \end{pmatrix} = \begin{pmatrix} 1-p & q \\ p & 1-q \end{pmatrix} \begin{pmatrix} S_n \\ H_n \end{pmatrix} \tag{2}$$

and the expected numbers of microbes on hands H and on surface S will approach a steady state. Wang et al. estimated the attachment and detachment coefficients q and p, given that steady state can be reached within a few touching events [1].

Fig 1 illustrated how the sequence of microbes on hands (NH) and microbes ingested (NI) are generated with hand touching and hand mouthing. Table 1 shows the attachement and detachment process and parameters used by behavior. And the details of the variables and parameters used in this paper can be found in Table 2

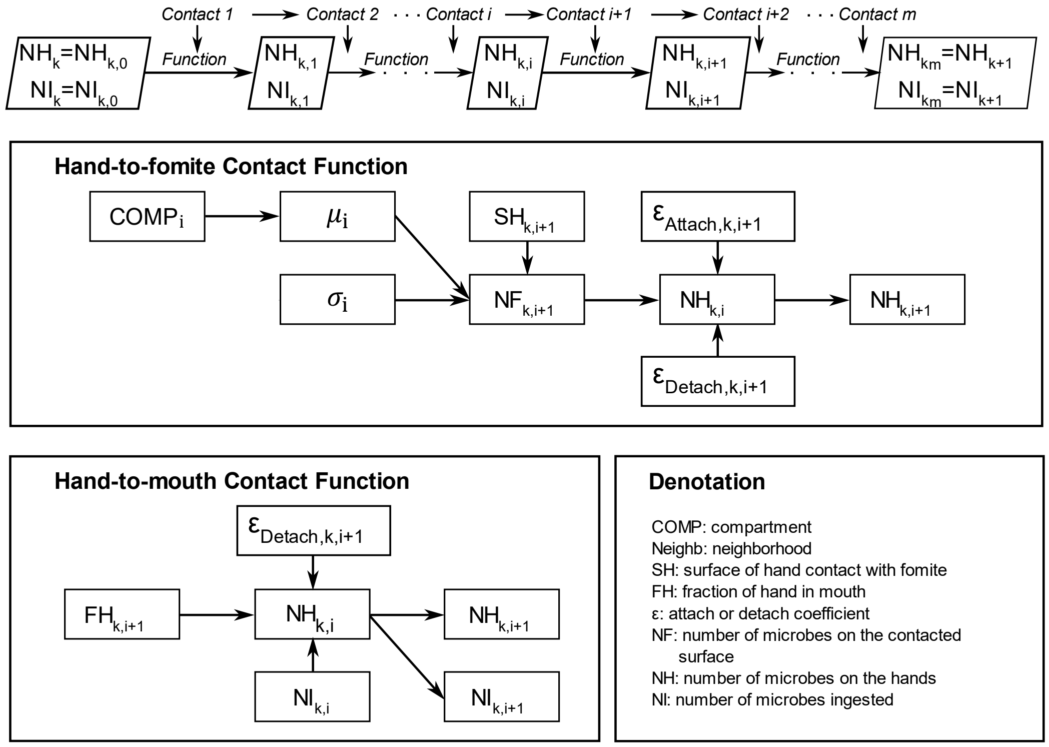

Fig 1. **Modules determined by behavior sequences of infant *k* calculate the microbes on hands (*NH*) and microbes ingested (*NI*).**

Table 1. **Process (attachment, detachment, ingestion) and parameters used for modules.**

Module	Process	Variables and Parameters
**Hand Touching**	attachment, detachment	log10 scale *E. coli* concentration of the surface contacted; attachment coefficient from surface to hand; detactment coefficient from hand to surface; area of hand touching the surface
**Mouthing**	detachment, ingestion	fraction of the surface of hands placed in mouth; fraction of microbes removed from hands by mouthing; area of fomites placed in mouth
**Bathing**	detachment	duration of bathing; fraction of microbes removed from hands by bathing; volume of bathing water ingested
**Pica**	attachment, detachment, ingestion	log10 scale *E. coli* concentration of soil; attachment coefficient from soil to hand; detactment coefficient from hand to surface; area of hand touching the surface; weight of soil ingested during pica
**Eating**	attachment, detachment, ingestion	log10 scale *E. coli* concentration of solid food; attachment coefficient from surface to hand; detactment coefficient from hand to surface; area of hand touching the surface; serving size of food
**Breastfeeding**	ingestion	log10 scale *E. coli* concentration of breastmilk; volume of breastmilk ingested
**Drinking Water**	ingestion	log10 scale *E. coli* concentration of water; volume of water ingested

Table 2. **Variables, parameters and distributions used in the model.**

Variables	Parameters	Distributions/Models	Probability Density Function/Formulas	Sources
Area of the hand touching the surface (cm^2) at Timepoint 1	$a = 0$ $b = 15.75$	Uniform	$f(x) = \begin{cases} \frac{1}{b-a} & \text{for } x \in [a, b] \\ 0 & \text{otherwise} \end{cases}$	Provided by Haramaya University
Area of the hand touching the surface (cm^2) at Timepoint 2	$a = 0$ $b = 29.25$	Uniform	$f(x) = \begin{cases} \frac{1}{b-a} & \text{for } x \in [a, b] \\ 0 & \text{otherwise} \end{cases}$	Provided by Haramaya University
Fraction of the surface of hands placed in mouth	$\alpha = 3.7$ $\beta = 25$	Beta	$f(x) = \frac{x^{\alpha-1}(1-x)^{\beta-1}}{B(\alpha, \beta)}$	Ozkaynak et al., Zartarian et al. [2, 3]
Attachment coefficient from surfaces to hands	$a = 0.01$ $b = 0.19$ $c = 0.1$	Triangle	$f(x) = \begin{cases} \frac{2(x-a)}{(b-a)(c-a)} & \text{for } a \le x < c \\ \frac{2(b-x)}{(b-a)(b-c)} & \text{for } c \le x \le b \\ 0 & \text{otherwise} \end{cases}$	Assumption
Detachment coefficient from hands to surfaces	$a = 0.25$ $b = 0.75$ $c = 0.5$	Triangle	$f(x) = \begin{cases} \frac{2(x-a)}{(b-a)(c-a)} & \text{for } a \le x < c \\ \frac{2(b-x)}{(b-a)(b-c)} & \text{for } c \le x \le b \\ 0 & \text{otherwise} \end{cases}$	Assumption
Duration of bathing (s)	$k = 4$ $\theta = 60$	Gamma plus 60s (constant value)	$f(x) = \frac{1}{\Gamma(k)\theta^k} x^{k-1} e^{-x/\theta}$	Assumption
Log10 fraction of microbes remaining after bathing (no soap)	$\beta_0 = 0.68$ $\beta_1 = -0.80$	Linear model with log(duration)	$f(x) = \beta_0 + \beta_1 log(x)$	Montville et al., Kampf et al. [4, 5]

Continued on next page

Continued from previous page

Variables	Parameters	Distributions/Models	Probability Density Function/Formulas	Sources
Fraction of microbes removed from hands by hand mouthing	$a = 0.01$ $b = 0.40$ $c = 0.33$	Triangle	$f(x) = \begin{cases} \frac{2(x-a)}{(b-a)(c-a)} & \text{for } a \le x < c \\ \frac{2(b-x)}{(b-a)(b-c)} & \text{for } c \le x \le b \\ 0 & \text{otherwise} \end{cases}$	Amadi et al. [6]
Area of fomite mouthed (cm^2)	$a = 10$ $b = 50$	Uniform	$f(x) = \begin{cases} \frac{1}{b-a} & \text{for } x \in [a,b] \\ 0 & \text{otherwise} \end{cases}$	Assumption
E. coli concentration (CFU/mg or CFU/mL or CFU/cm^2)	k varies by sample type θ varies by sample type & neighborhood	Gamma	$f(x) = \frac{1}{\Gamma(k)\theta^k} x^{k-1} e^{-x/\theta}$	Estimate from environmental sample data
Probability of drinking breastmilk given drinking behavior for infant at Timepoint 1	$\alpha = 1173$ $\beta = 247$	Beta	$f(x) = \frac{x^{\alpha-1}(1-x)^{\beta-1}}{B(\alpha,\beta)}$	Estimate from structured observation data
Probability of drinking breastmilk given drinking behavior for infant at Timepoint 2	$\alpha = 868$ $\beta = 305$	Beta	$f(x) = \frac{x^{\alpha-1}(1-x)^{\beta-1}}{B(\alpha,\beta)}$	Estimate from structured observation data
Volume of bathing water ingested (in mL) during bathing for infants at Timepoint 1	$a = 0$ $b = 2.69$	Uniform	$f(x) = \begin{cases} \frac{1}{b-a} & \text{for } x \in [a,b] \\ 0 & \text{otherwise} \end{cases}$	USEPA [7]

Continued on next page

Continued from previous page

Variables	Parameters	Distributions/Models	Probability Density Function/Formulas	Sources
Volume of bathing water ingested (in mL) during bathing for infants at Timepoint 2	$a = 0$<br>$b = 1.37$	Uniform	$f(x) = \begin{cases} \frac{1}{b-a} & \text{for } x \in [a, b] \\ 0 & \text{otherwise} \end{cases}$	USEPA [7]
Volume of breastmilk ingested (in mL) during a breastfeeding event for infants at Timepoint 1	$a = 32.7$<br>$b = 54.5$	Uniform	$f(x) = \begin{cases} \frac{1}{b-a} & \text{for } x \in [a, b] \\ 0 & \text{otherwise} \end{cases}$	USEPA [7]
Volume of breastmilk ingested (in mL) during a breastfeeding event for infants at Timepoint 2	$a = 54.5$<br>$b = 68.125$	Uniform	$f(x) = \begin{cases} \frac{1}{b-a} & \text{for } x \in [a, b] \\ 0 & \text{otherwise} \end{cases}$	USEPA [7]
Volume of water ingested (in mL) during a drinking water event for infants at Timepoint 1	$a = 32.7$<br>$b = 54.5$	Uniform	$f(x) = \begin{cases} \frac{1}{b-a} & \text{for } x \in [a, b] \\ 0 & \text{otherwise} \end{cases}$	USEPA [7]
Volume of water ingested (in mL) during a drinking water event for infants at Timepoint 2	$a = 54.5$<br>$b = 68.125$	Uniform	$f(x) = \begin{cases} \frac{1}{b-a} & \text{for } x \in [a, b] \\ 0 & \text{otherwise} \end{cases}$	USEPA [7]
Serving size of food (g)	50	constant		Assumption
Weight of soil ingested (g) per pica event	1.25	constant		Assumption

Key Assumptions

In order to keep the model manageable, we made several simplifying assumptions, which are listed below to clearly define the constraints of the model:

1. All simulated child days started with the state "sleeping down on a surface with barriers" (i.e., sleeping on the bed). The length of a child-day (daytime period) was 14 hours and we considered exposure outside the daytime period trivial.
2. Similar as in Wang et al., the coefficient of detachment was high, and larger than the coefficient of attachment, so that repeated touching of a contaminated surface rapidly resulted in stable numbers of microbes on hands [1, 8].
3. The die-off rate of microbes was set to zero, which means that the number of viable microbes was not assumed to decrease during the time they were attached to hands.
4. Although only a small area of a hand has contact with fomites and enters the mouth during hand mouthing, we assumed that all microbes present on hands after a contact event were instantaneously redistributed on the skin of the hand, so that their surface density remained uniform.
5. Many parameters in the model are based on our best knowledge from literature, discussions with local partners, and assumptions. Wang et al. conducted a validation study using handrinse data of children and found that the simulated and observed hand contamination were within a similar magnitude [1].

References

[1] Wang Y, Moe CL, Teunis PF. Children are exposed to fecal contamination via multiple interconnected pathways: a network model for exposure assessment. Risk Analysis. 2018;38(11):2478–2496. doi:10.1111/risa.13146.

[2] Özkaynak H, Xue J, Zartarian VG, Glen G, Smith L. Modeled estimates of soil and dust ingestion rates for children. Risk Analysis: An International Journal. 2011;31(4):592–608. doi:10.1111/j.1539-6924.2010.01524.x.

[3] Zartarian VG, Xue J, Özkaynak H, Dang W, Glen G, Smith L, et al. A probabilistic arsenic exposure assessment for children who contact CCA-treated playsets and decks, Part 1: Model methodology, variability results, and model evaluation. Risk Analysis: An International Journal. 2006;26(2):515–531. doi:10.1111/j.1539-6924.2006.00747.x.

[4] Montville R, Chen Y, Schaffner DW. Risk assessment of hand washing efficacy using literature and experimental data. International journal of food microbiology. 2002;73(2-3):305–313. doi:10.1016/S0168-1605(01)00666-3.

[5] Kampf G, Kramer A. Epidemiologic background of hand hygiene and evaluation of the most important agents for scrubs and rubs. Clinical microbiology reviews. 2004;17(4):863–893. doi:10.1128/cmr.17.4.863-893.2004.

[6] Amadi EC, Nwagu TN, Emenuga V. Mobile phones of health care workers are potential vectors of nosocomial agents. Afr J Microbiol Res. 2013;7(22):2776–2781. doi:10.5897/AJMR12.2353.

[7] U S Environmental Protection Agency (EPA). Exposure factors handbook : 2011 edition. National Center for Environmental Assessment, Washington, DC; 2011.

[8] Wang Y, Moe CL, Null C, Raj SJ, Baker KK, Robb KA, et al. Multipathway quantitative assessment of exposure to fecal contamination for young children in low-income urban environments in Accra, Ghana: the SaniPath analytical approach. The American Journal of Tropical Medicine and Hygiene. 2017;97(4):1009. doi:10.4269/ajtmh.16-0408.
