# Supplementary material for: Quantitative multi-pathway assessment of exposure to Escherichia coli for infants in Rural Ethiopia
Source: PLoS Negl Trop Dis. 2025 Jun 9;19(6):e0013154. doi: 10.1371/journal.pntd.0013154 (PMC12176293; doi:10.1371/journal.pntd.0013154)
Supplement: S1 Table — For the areola swab, fomites, handrinse, and soil samples, we reported elution volume as collection volume. The approximate surface area of bootsock samples is between 8000 and 10000 cm2. (PDF) [file pntd.0013154.s013.pdf]

Table S1. **Lab methods for different sample types.** For the areola swab, fomites, handrinse, and soil samples, we reported elution volume as collection volume. The approximate surface area of bootsock samples is between 8000 and 10000 cm^2.

Sample Type	Collected Volume	Detection Method	Detection Volume	Limit of Detection
Areola Swab	swab (4 mL)	EC MUG	5 wells of 180 uL	4.17 MPN/swab
Fomites	Sponge (5 mL)			3.70 MPN/sponge
Handrinse	pair of hands (200 mL)	EC MUG	88 wells of 180 uL	12.63 MPN/pair of hands
Bathing Water	1 L			0.06 MPN/mL
Drinking Water	1 L			0.06 MPN/mL
Food	1–10 g	EC MUG or Chromocult	5 wells of 180 uL	3.75 CFU/gram
Breast Milk	0.5–5 mL	Chromocult	200 uL with serial dilutions	5 CFU/mL
Soil	Boot socks (15 mL)			75 CFU/boot sock
