# Supplementary material for: Quantitative multi-pathway assessment of exposure to Escherichia coli for infants in Rural Ethiopia
Source: PLoS Negl Trop Dis. 2025 Jun 9;19(6):e0013154. doi: 10.1371/journal.pntd.0013154 (PMC12176293; doi:10.1371/journal.pntd.0013154)
Supplement: S2 Table — (PDF) [file pntd.0013154.s014.pdf]

Table S2. **Estimated parameters of normal distributions for log 10 scale *E. coli* concentrations at two timepoints.**

	Timepoint 1		Timepoint 2		Total		
Sample Type	Mean	SD	Mean	SD	Mean	SD	Unit
Areola Swab	-0.043	0.674	-0.434	1.141	-0.287	0.945	per swab
Breast Milk	-0.203	1.103	-1.573	1.197	-0.822	1.218	per mL
Mother Handrinse	2.051	1.070	1.947	0.981	2.000	1.029	per pair of hands
Sibling Handrinse	2.029	0.926	2.001	0.924	2.020	0.925	per pair of hands
Infant Handrinse	1.145	0.941	1.885	1.074	1.490	1.101	per pair of hands
Bathing Water	-0.324	1.093	-0.420	0.975	-0.371	1.045	per mL
Drinking Water	-0.709	0.727	-1.176	0.714	-0.944	0.758	per mL
Fomite	0.765	0.864	0.680	1.483	0.725	1.210	per sponge
Food	-0.012	1.570	-0.658	1.681	-0.436	1.663	per gram
Soil			5.188	0.866	5.188	0.866	per boot sock
