# Supplementary material for: Quantitative multi-pathway assessment of exposure to Escherichia coli for infants in Rural Ethiopia
Source: PLoS Negl Trop Dis. 2025 Jun 9;19(6):e0013154. doi: 10.1371/journal.pntd.0013154 (PMC12176293; doi:10.1371/journal.pntd.0013154)
Supplement: Attachment — Submitted filename: Response to Reviewers.docx [file pntd.0013154.s015.docx]

**All the replies were shaded and line numbers are based on the manuscript with tracked changes version.**

**Reviewers' Comments:**

Reviewer's Responses to Questions

**Key Review Criteria Required for Acceptance?**

As you describe the new analyses required for acceptance, please consider the following:

**Methods**:

-Are the objectives of the study clearly articulated with a clear testable hypothesis stated?

-Is the study design appropriate to address the stated objectives?

-Is the population clearly described and appropriate for the hypothesis being tested?

-Is the sample size sufficient to ensure adequate power to address the hypothesis being tested?

-Were correct statistical analysis used to support conclusions?

-Are there concerns about ethical or regulatory requirements being met?

Reviewer #1: Goals of the study are clearly stated, though they seem to overstate what the study was able to accomplish in that comprehensive characterization of behavior is challenging from only two time points and in that the quantification of exposure to fecal contamination is difficult from indicator bacteria given the limitations of said indicator.

**Reply:** We reframed the paper and clearly explained usage of E. coli in this study. And we replaced “fecal contamination” with *E. coli* throughout the paper to make it more clear.

Ethiopian specific vocabulary needs to be defined (i.e., kebeles, injera, etc.) in order to cater to a larger audience.

**Reply:** Done. See lines 120-121 and 460.

Sample size is strong, though the authors need to address the lack of soil data from time point 1.

**Reply:** The focus of this study is to understand the difference between exposures of infants at two developmental phases driven by their behavior changes instead of comparing *E. coli* contamination at different periods. As this study was based on a rolling enrollment, the soil samples at Timepoint 2 were collected between December 2021 and June 2022, which covered part of the seasonality and temporal variation. In lines 167-168, we added “Soil samples were only collected at Timepoint 2 as we observed a limited mobility and contact with soil of infants at Timepoint 1 in our pilot study.”

The decision to consider drinking a duration behavior compared to eating as a frequency-based behavior needs justification. Samples are listed as clear liquids that should not be considered clear liquids.

**Reply:** Our exposure assessment studies (EXCAM and SaniPath) recorded the most common (with the most time spent) ingestive behaviors as duration-based observations. In the 2012 SaniPath study in Ghana (Wang et al., 2017), the eating behavior (for solid food) was recorded as duration-based observations (with start and end times) for children under five. For infants in this study, the most common (and probably most important) ingestive behavior was drinking breastmilk (breastfeeding) and eating solid food was rare based on conversations with stakeholders during the pilot study phase, which informed our study design. In lines 152-155, we added “Drinking, mostly breastfeeding, was recorded as a duration-based behavior as it is the most common ingestive behavior (with most time spent) for infants while rare instances of eating solid food was recorded as a frequency-based behavior.”

We also revised the sentence in line 172-173 related to liquid extracted from samples as “EC MUG, a fluorometric approach, was used for drinking water, bathing water, handrinse, and the liquid extracted from the fomites and areola swabs.”

While a previous publication is referenced, the author still needs to provide a more thorough overview of methodology including the limitations cited in the external paper (i.e., sample volume, lack of sterilization of caregiver hands prior to breast milk collection, pathogenic vs. nonpathogenic E. coli, etc.). In the other study, it references positive controls but not negative controls or the results of said controls. This needs to be included in the work.

**Reply:** We provided a brief overview of the lab methodology in lines174-187.

Would appreciate a discussion of whether or not seasonality played a role in environmental contamination risks.

**Reply:** After careful consideration, we think this paper does not have enough data to discuss the seasonality of environmental contamination risk as our sample collection was based on a rolling enrollment design. However, based on our previous experience, exposure to fecal contamination can be driven by seasonality. For example, in Cambodia, during the rainy season, the contact frequency of flood water increases dramatically for residents in the city as they just cannot avoid the flood water on the street. On the other hand, the fecal contamination concentration in the flood water could decrease with high dilution by rainwater or sometimes increase with overflowed drains. We added a sentence in the limitation (lines 454-456) to explain that this study could not examine this important topic (i.e., seasonality): “Also, our environmental sample collection (based on a rolling enrollment) cannot systematically examine the environmental contamination risk driven by seasonality which has been reported by previous studies (Ronoh et al., 2020; Mwapasa et al., 2024).”

The authors should provide justification for the use of a normal distribution for concentration data. Did they carry out statistical analysis on their human/environmental sampling data to determine this?

**Reply:** In this study, we used the lognormal distribution for the *E. coli* concentration (or normal distribution for log10 *E. coli* concentration). In the 2012 SaniPath Accra study (Wang et al., 2017), we used the membrane filtration method resulting in counts of colonies. During that time, the count data was modeled as a Poisson distribution, and the E. coli concentration was a Poisson gamma mixture (following negative binomial distribution). However, different lab methods (e.g., IDEXX) were used in later SaniPath studies. In environmental samples from those SaniPath studies and this EXCAM, the *E. coli* concentrations were usually right-skewed with long tails. After the log transformation, we examined the data visually using boxplots (e.g., Fig 2 in Wang et al., 2022) and qqplots. We also conducted statistical tests for normality such as the Shapiro-Wilk test (almost all failed to reject the null hypothesis).

The authors need to include the calculations for exposure, including whether or not transfer efficiencies were used, how surface areas of touching and mouthing behaviors were considered, how were timeseries behaviors accounted for, etc.

**Reply:** We agree that more details of the methods must be included. The exposure was calculated based on the simulated behavioral sequence and estimated *E. coli* levels of different environmental samples using the modules we created. We included the details of the exposure model, parameters, and assumptions (2 additional supplementary tables and 1 additional supplementary figure) in the Supplementary Material.

Reviewer #2: 1. The methods lack the clarity required to repeat the study. While the details may be in the github code, it should not be the responsibility of the reader to comb through the code to determine critical information such as the transfer efficiencies used in the modules to determine the concentration of E. coli on the hand before and after specific transitions.

**Reply:** As described in the previous reply, we included the details of the exposure model, parameters, and assumptions (2 additional supplementary tables and 1 additional supplementary figure) in the Supplementary Material.

2. The methods also exclude critical details of the modules such as soil not being assessed at Timepoint 1 and direct contact with feces not included at Timepoint 1 or Timepoint 2. The authors make strong conclusions that food and breastfeeding as primary pathways in Timepoint 1 without acknowledging that soil could be a primary pathway but it was not considered at Timepoint 1.

**Reply:** The soil pathway was assessed at Timepoint 1 using behavior data from Timepoint 1 and soil environmental sample data at Timepoint 2. As we explained to Reviewer #1, the focus of this study is to understand the difference between exposures of infants at two developmental phases driven by their behavior changes instead of comparing *E. coli* contamination at different periods. As this study is based on a rolling enrollment, the soil samples at Timepoint 2 were collected between December 2021 and June 2022, which covered part of the seasonality and temporal variation. In lines 167-168, we added “Soil samples were only collected at Timepoint 2 as we observed a limited mobility of infants at Timepoint 1 in our pilot study.” In lines 316-318, we added “As we did not collect soil samples at Timepoint 1, the estimated parameters from soil samples at Timepoint 2 were used for the exposure assessment at both Timepoint 1 and Timepoint 2.”.

3. The methods section on the modules should also explain how the frequency of events was translated into the quantity of food/soil ingested. Given that the E. coli was measured as concentration per volume, it is necessary to estimate the volume of food/soil ingested to estimate the quantity of E. coli ingested. The quantity of E. coli ingested cannot be estimated from the frequency alone. Without a valid methodology for this conversion, the method cannot be considered valid.

**Reply:** As described in the previous reply, we included the details of the exposure model, parameters, and assumptions (2 additional supplementary tables and 1 additional supplementary figure) in the Supplementary Material.
4. Figure 2: There is an arrow from NI(i) to NI(i+1) but it is not clear why NI(i) impacts NI(i+1). This aspect of the model should be explained in the methods.

**Reply:** *NI* is a sequence that tracks cumulative ingestion. Therefore, *NI(i+1)* = *NI(i)* + Ingestion Generated by Module. This has also been clarified in the new supplementary section for modeling details.

5. Given the potential importance of direct fecal ingestion, the authors should justify why this activity was not included in the list of observed behaviors.

**Reply:** The “direct contact with feces” was not dropped but reformatted under “pica” as one of the subcategories, “pica - feces” (see Table 2). In this study, “pica” was defined as an event when any of the non-edible items is placed into the mouth such that it could be swallowed: feces, soil, or other. We also added this definition in the Method Section. However, the pica-feces was rare, so we focused on pica-soil in this study and did not collect environmental samples to match this behavior.

6. It appears that the behavioral categories and compartments used are not comprehensive and mutually exclusive. This both confuses the presentation and raises questions about what is being modeled. For example, Table 1 presents the percentage of time awake, bathing, drinking, and sleeping. Children should be awake while bathing and drinking, so the percentage of time awake should be inclusive of the time bathing and drinking. One way to resolve this is to describe the time “awake” as the time “playing” or “awake but not bathing and drinking”. Table 1 also lists the compartments of “carried by mother”, “carried by other”, “on the bare ground”, and “on a surface with barriers”. How is the time classified when the child is awake but on a bed or chair? Awake but on a mat? What is the definition of a “surface with barriers”? Furthermore, why was “touching soil” not included? If the model seeks to represent the E. coli contamination on hands, then the frequency of touching soil indoors on a mat, indoors on the ground, and outdoors should be included in the model, along with the respective E. coli on these surfaces (instead of just one concentration for E. coli on “soil”). Having lists of activities and compartments that are comprehensive and mutually exclusive is essential given the desire to use a competing hazard model.

**Reply:** The behavioral categories and compartment categories were determined after our pilot observations. We appreciate the reviewer’s comment about clarifying the awake behavior to ensure the behaviors are mutually exclusive. We added “To ensure the duration-based behaviors are mutually exclusive, the "awake" behavior in this study was considered as awake but not bathing or drinking” in lines 151-152. The behavior, compartment, and location sequences are paralleled (see Supplementary Figures 5 and 7). So the child can be in one of the states, defined as combinations of behavior, compartment, and location (e.g., awake, on a bed, on homestead), at a specific time point. Thanks for pointing out that the definition of “surface with barriers” is missing. We added “The “barriers" refers to the covering (e.g., a mat or blanket) put between the infant and surface (e.g., ground, bed) when the infant is laying down without the assistance of a person.” in line 158-159. Touching soil was considered a special case of “touching fomites” when the infant is “on the bare ground”. In the pilot data collection, it was very challenging to record separate touching fomites, touching different surfaces, and touching grounds behavior. Therefore, we decided to simplify this, including all those touching behaviors as “touching fomites” and using compartments to link with specific environmental samples. For example, soil samples linked with “touching fomites” + “on the bare ground”, while fomites samples linked with “touching fomites” + “on a surface with barriers”. Based on limited resources, the soil samples were only collected from outside the household and we did not distinguish indoor and outdoor soil samples.

7. The authors assumed that children were awake for 14 hours at both Timepoint 1 and Timepoint 2. Instead, they should use empirical data (from their study or others) to estimate the actual time awake. Other studies have found that as children age, they sleep less.

**Reply:** We assumed 14 hours of daytime (as our observation occurred during the day), not 14 hours “awake” time, in the simulations. Please refer to the sentence in lines 255-256: “In this study, we generated microbe transfers during 10,000 typical child days (14 hours daytime assumed) for each timepoint.” The “sleeping” behavior was simulated based on parameters estimated from our collected behavior sequence data by timepoint. Therefore, as children sleep less at timepoint 2, we will see less proportion of the 14 hours occupied by “sleeping” in the simulations.

8. What does “pica – other” include? - any non-edible (non-food time)

**Reply:** We had a main/primary key (button) for pica on our mobile data collection app, then they could specify a secondary key, whether it was feces, soil, or other (anything other than those two). As mentioned in the previous replies, “pica” was defined as an event when any of the non-edible items is placed into the mouth such that it could be swallowed: feces, soil, or other.

9. Figure 4: The authors should describe in the methods why they consider food to be a vector instead of a source and how this affects the model. Since we know that contamination in food can grow over time, the authors should also consider a sensitivity analysis that treats the food as a source instead of a vector. Similarly but likely less importantly, bathing water should be considered as a source in some cases – e.g. a drop of bathing water placed in the mouth of an infant is acting as a source, whereas the child’s skin going into the bathing water has the water acting as a sink.

**Reply:** The food was considered as a vehicle and source at the same time. In the simulation, it was formatted as a vector of numbers tracing contamination from different sources including itself. Please refer to the detailed exposure model method we added in the Supplementary Materials A. For bathing water, we did consider it as sink and source at the same time. You can see small red arrows from Bathing Water to Mouth. However, we decided to color it green as it is predominately act as a sink in this case. We added a sentence “Food is considered as a *vehicle* and *source*, while bathing water is considered as a *sink* and *source*.” in the caption of Figure 4 to clarify the roles of food and bathing.

10. Lines 99-101: Explain how many local enumerators there and what standard of inter-rater reliability they had to achieve before the started collecting data. During data collection how often was inter-rater reliability re-assessed and what did you do with data that did not meet the standard?

**Reply:** We had four enumerators. We used an interobserver agreement, not interrater reliability (the enumerators weren’t rating anything). We had the enumerators trained to 80% agreement in the natural environment (in infants’ home) with a trained observer before starting data collection. We conducted three scheduled training sessions and provided additional training whenever needed. We added those details in the lines 140-144.

Reviewer #3: Yes, I suggest additional clarity on some statistical test results described, and also some more statistical tests suggested.

**Reply:** We added the statistics tests in the methods and table captions as well as p-values whenever presenting significant differences for both behavior and environmental sample results.

**Results**:

-Does the analysis presented match the analysis plan?

-Are the results clearly and completely presented?

-Are the figures (Tables, Images) of sufficient quality for clarity?

Reviewer #1: In lines 197-199, the sentence structure needs to be re-written to be clear what the percentage is specifying. As currently written, it very unclear what the percentage refers to.

**Reply:** We rewrote the sentence “Bathing behavior was infrequent and short for both timepoints. Only 30.4% of study infants at Timepoint 1 and 57.9% at Timepoint 2 bathed during our observations. And the average length of bathing is 5.8 mins at Timepoint 1 and 2.1 mins at Timpoint 2.”

When discussing correlations, the author should address strength of correlation via numerical values.

**Reply:** We added Spearman’s rho for all the correlations to address their strength.

The author needs to not refer to positive E. coli samples as fecal contamination. Not all E. coli is fecal contamination and much of the E. coli found in the environment is non-pathogenic. The author should use specific language to specify either E. coli contamination or an indication of possible fecal contamination.

**Reply:** Agreed. We revised this throughout the paper, changing fecal contamination to either *E. coli* or fecal indicator.

It feels that the results section could be more profound. Some of the results section was previously reported, making the portion that is newly explored minimal. Are there are other ways the author could look at the data to provide a more robust set of results? For example, looking at cumulative exposure over a given day (or other time period) in addition to just exposure pathways.

**Reply:** For the exposure assessment results, we typically examine the relative contribution by pathway (Figure 3), cumulative exposure (i.e., daily dose) over a given day by pathway (Figure S12), and fecal microbe transfer network (Figure 4). For this paper, we focused on adapting our models and comparing more complex behavior data and exposure results. We have reported the time course of exposure to E. coli by pathway and dominant pathway for 100 simulated days in Figure 5 (see below) of Wang et al., (2018). However, to better describe individual exposure over a long period of time, we need to develop a behavior sequence model at individual level instead of our current neighborhood level model. This could be out of the scope of this paper.

Wang, Y., Moe, C. L., & Teunis, P. F. (2018). Children are exposed to fecal contamination via multiple interconnected pathways: a network model for exposure assessment. *Risk Analysis*, *38*(11), 2478-2496.

The last sentence of the results section seems to be contradicted by the data and the discussions section with the environment (soil) - hand - mouth pathway being substantially important at time point 2.

**Reply:** In this study, we considered direct pathways (source to mouth) and indirect pathways (source to vehicle to month). Direct pathway and indirect pathway can happen at the same time during a behavior such as pica-soil. In Figure 4, the arrow pointing from soil to mouth represents the direct pathway part of exposure and the arrow pointing from “soil => hand => mouth” and “soil => hand => food => mouth” represent the indirect pathways. In the last sentence, we are talking about the indirect pathway part is relatively small (we revised the sentence to clarify this). And in the discussion, we are referring to the direct pathway part of pica soil led to high exposure. The reason we separate the direct and indirect pathways in our exposure quantification is to provide evidence for more prescriptive intervention recommendations. Using soil pathway as an example, as indirect pathways can be impacted by hand and food hygiene interventions, the direct pathway are impacted by behavioral change interventions or education. We appreciate the reviewer pointing this out and we clarified this “direct” vs “indirect” pathways in the Methods section under the Multi-pathway Exposure Assessment subsection.

Reviewer #2: 1. Table 1 should include the percentage of children who could crawl, since crawling strongly influences mouthing and touching behaviors.

**Reply:** We do have some data on crawling but only for half of the participants at Timepoint 2. It was 100% of them. Crawling information got brought up (the enumerators left a note saying if they were crawling or walking) and added to the behavior data collection after we started data collection at the Timepoint 2.

2. Figure 3 contains several boxes that are not labeled – what are these?

**Reply:** Those boxes are other source that did not contribute substantially (<1%) to the total exposure of infants. The treemap is a type of plot presenting the part to whole relationships. It visualizes the quantities and patterns of different categories in order. The treemaps here are used to simply the exposure results and highlight the “top contributors” to the infant exposure. Therefore, those small boxes are trivial information to show in this figure. However, we did present the exposure to *E. coli* by source and timepoint in the Supplementary Figure 12 as boxplots and barcharts.

3. Table S1 is misleading and not clear. Firstly, what does “collection volume” mean? The areola sample was collected with a swab. Does “collected” volume mean “elution volume? Secondly, the table states that up to 1 L of sample was processed, but Deblais 2024 reports that a maximum of 15.84 mL of sample was processed. This results in a much lower limit of detection than would be expected by the reader given the 1 L of sample collected. It is unclear why 1 L of sample was collected when only 15 mL was assays (especially given the limited availability of drinking water at households), or, conversely, why so little sample was assayed without prior concentration of the sample. To clarify, Table S1 should include the lower limit of detection in MPN and CFU. Table 1 should also note the approximate surface area represented by the sample (e.g. for bootsocks, what is the approximate m2 that was covered with “15 steps” (Deblais 2024). The table (and methods section) should also specify is the “soil” referred to in the paper is from inside or outside of the households, as Deblais 2024 notes that three soil samples were collected, two from inside and one from outside. Presenting the surface areas represented will help determine the load, allowing for comparison to other studies.

**Reply:** We thank the reviewer for pointing out that Table S1 is unclear. We explained in the caption that for the areola swab, fomites, handrinse, and soil samples, we reported elution volume as collection volume. We confirmed that 1 L of drinking water and bathing water were collected, as those samples were also used to test for Campylobacter. We added the limit of detection to the table as the reviewer suggested. We added a sentence to the caption explaining that the approximate area of bootsock samples is between 8000 and 10000 cm2.

4. Figure S4 – the results state that children commonly took naps at noon at Timepoint 2 but this is not apparent from Figure S4.

**Reply:** We feel it is quite clear that the yellow strips are around noon time at Timepoint 2. To provide better visuals for the reviewers, we plotted the average proportion of time spent for different duration-based behaviors among our study infants by timepoint (see below).

Reviewer #3: Yes, I provide some details where additional information is suggested.

**Conclusions**:

-Are the conclusions supported by the data presented?

-Are the limitations of analysis clearly described?

-Do the authors discuss how these data can be helpful to advance our understanding of the topic under study?

-Is public health relevance addressed?

Reviewer #1: The discussion (and conclusions) is supported by the data present (with the exception of the last sentence of results addressed above in the Results section).

**Reply:** We clarified the last sentence of the results in our reply above.

The studies for comparison to support frequent geophagia (soil pica) were not strong support.

**Reply:** In the paper, we did not indicate that the soil-pica behavior is frequent. We stated “that soil-pica is not a rare behavior among young children”, which means that soil-pica should not be ignored, based on the following studies:

1. Calabrese, E.J., Stanek, E.J., 1993. Soil pica: not a rare event. Journal of Environmental Science & Health Part A 28, 373–384. doi:10.1080/10934529309375884.
2. Moya, J., Phillips, L., 2014. A review of soil and dust ingestion studies for children. Journal of Exposure Science & Environmental Epidemiology 24, 545–554. doi:10.1038/jes.2014.17.
3. Humphrey, J.H., Mbuya, M.N., Ntozini, R., Moulton, L.H., Stoltzfus, R.J., Tavengwa, N.V., Mutasa, K., Majo, F., Mutasa, B., Mangwadu, G., Chasokela, C.M., Chigumira, A., Chasekwa, B., Laura E Smith, L.E., Tielsch, J.M., Jones, A.D., Manges, A.R., Maluccio, J.A., Prendergast, A.J., for the Sanitation Hygiene Infant Nutrition Efficacy (SHINE) Trial Team, 2019. Independent and combined effects of improved water, sanitation, and hygiene, and improved complementary feeding, on child stunting and anaemia in rural Zimbabwe: a cluster-randomised trial. The Lancet Global Health 7, e132–e147. doi:10.1016/S2214-109X(18)30374-7.

Those studies were chosen as they focused on children in LMICs. We added another study to support this case:

Shivoga, W.A., Moturi, W.N., 2009. Geophagia as a risk factor for diarrhoea. The Journal of Infection in Developing Countries 3, 094–098. doi:10.3855/jidc.55

The authors should elaborate on the impacts of the missing observation time. Are there thoughts on this time being higher or lower impact for exposure? All behavior is somewhat limited by observation time because behaviors cannot be observed at all times, so this limitation should be caveated as minor, observations were long.

**Reply:** As the reviewer mentioned, our observations were long, from 8 AM to 6 PM during the day. As we observed in this study, infants’ behavior patterns are less impacted by the time of the day and infants spend almost all the time within the household. For exposure assessment, we assumed the behavior pattern unobserved after 6 PM is similar to that between 8 AM to 6 PM. Then, we estimated the parameters from our observation and simulated the behavior sequence for 14 hours, which goes beyond our observation period. Based on our current knowledge, we have not identified any behaviors that could increase or decrease the exposure dramatically during the unobserved period. However, we agree that unobserved behavior could lead to potential underestimation or oversight in exposure. We did have a sentence elaborating this:

“Also, we were not able to observe the infants during specific time periods, such as right after waking up in the morning or before going to sleep at night. This could lead to potential underestimation or oversight in identifying the contributions from specific pathways to exposure to fecal contamination and enteric pathogens.”

in the limitation paragraph in lines 438-441.

In the Conclusions, the month range is specified more clearly. This should be in methods.

**Reply:** Agree. We revised the month range in the method as “the first round occurred when infants were 4–8 months old (Timepoint 1), and the second round occurred when they were 11–15 months old (Timepoint 2).”

The authors need to address other limitations of the study (which were alluded to above) - especially the idea of indicator organisms not necessarily being pathogenic and the role of environmental reservoirs of nonpathogenic E. coli. Another limitation that might be worth discussing is the idea of multi-pathogen exposure that cannot be captured by only one indicator organism.
Support the public health relevance for interventions and the future research into more specific enteric pathogens.

**Reply:** In previous SaniPath study, we have discussed the limitation of using fecal indicator and that’s one of the reasons we test pathogen in our later studies. In this study, we did test the samples for Campylobacter. As this paper is more focused on the behavior and exposure model. We believe it will be more appropriate to discuss the association between fecal indicator and specific pathogen as well as the specific pathogen intervention based on the Campy results in a separate paper.

Reviewer #2: The conclusions are not specific to the data, but rather seem speculative / based on the results of other papers.
Some specific comments on the intro and discussion section:
1. The discussion could be stronger by comparing the frequency of activities with those found in prior time-activity studies in LMICs and high-income countries. To facilitate this comparison, the rate of any drinking, any eating, any mouthing, and any pica should be listed in Table 2 and presented in Figure S1.

**Reply:** As much as we would like to compare the behavior data between this study with our previous studies, a direct comparison is challenging. We only collected behavior sequence data (with behavior and compartment) in Accra, Ghana (2012), Kolkata, India (2018, not published), and Haramaya, Ethiopia (2022). The study design, including subjects, settings, behavior definitions, training, and data collection tool, has evolved over time makes comparing statistics like frequency of certain behavior without careful adjustment become not appropriate. For example, the enumerators are still using paper-based observation, which can only capture time in minutes, in the Ghana study, while we used mobile data collection tools to enable higher resolution (in seconds) in time of observation in India (using ODK) and Ethiopia (using Countee). And we only collected some frequency-based behavior such as touching and mouthing in thie Ethiopia study. We believe this is out of the scope of this paper. We plan to work on another paper to carefully compare the behavior information while acknowledging the differences caused by different study designs.

2. The discussion could also be improved by more specifically discussing data that is found in the results and not discussing data that is not found in the results. For example, the discussion makes some mention of the need for animal feces management, but presents no data that the soil is contaminated by poorly managed animal feces and does not refer to the literature from some studies that have found outdoor soil contamination to be associated with the ownership of free-roaming animals (e.g. Ercumen 2017 – animal feces contribute to domestic contamination)

**Reply:** We appreciate reviewer pointing this out and we revised the sentence and cited Ercumen et al. 2017 to support it in lines 410-411.

3. The concentration of touching fomites seems low compared to prior studies. The discussion should note why the authors think this was found.

**Reply:** If I understand this comment correctly, the reviewer is asking why the frequency of touching fomites seems low compared to the prior SaniPath studies. In the prior SaniPath study in Accra, Ghana, we did not collect any behavior data on touching fomites or surfaces. The frequency of hand touching surfaces in the simulation model was based on partially Freeman (2001) and our assumption (see Wang et al., 2018). Kwong et al. (2016) reported the median frequency of object mouthing for less than 1 year old is more than 20 times. However, the definition of behavior events is very different between studies.

We think the longer time that infants were carried by mother or other (about 55% of time) at Timepoint 1 compared to at Timepoint 2 (about 34% of time) plus the limited mobility when they were little were the causes for much lower frequency of touching fomites for infants at Timepoint 1. When the infant was carried by the mother or other, the frequency of touching was less than 3 times per hour at Timepoint 1 and less than 6 times per hour at Timepoint 2. But when they were not carried, the frequency of touching can be 10–20 times per hour at Timepoint 1 and 30 times per hour at Timepoint 2. We added one sentence to explain this in the discussion, at line 371-373: “We found a lower frequency of touching fomites for young infants at Timepoint 1 compared to Timepoint 2. This could be caused by the limited mobility of infants and longer time carried by mother or other at Timepont 1.”

Freeman, N. C., Jimenez, M., Reed, K. J., Gurunathan, S., Edwards, R. D., Roy, A., … Lioy, P. J. (2001). Quantitative analysis of children's microactivity patterns: The Minnesota children's pesticide exposure study. *Journal of Exposure Analysis and Environmental Epidemiology*, 11(6), 501–509.

Wang, Y., Moe, C. L., & Teunis, P. F. (2018). Children are exposed to fecal contamination via multiple interconnected pathways: a network model for exposure assessment. *Risk Analysis*, 38(11), 2478-2496.

Kwong, L. H., Ercumen, A., Pickering, A. J., Unicomb, L., Davis, J., & Luby, S. P. (2016). Hand-and object-mouthing of rural Bangladeshi children 3–18 months old. *International journal of environmental research and public health*, 13(6), 563.

Reviewer #3: (No Response)

**Editorial and Data Presentation Modifications?**

Use this section for editorial suggestions as well as relatively minor modifications of existing data that would enhance clarity. If the only modifications needed are minor and/or editorial, you may wish to recommend “Minor Revision” or “Accept”.

Reviewer #1: See above comments. In addition to the above comments, the author should reconsider the title because it is misleading to specify fecal contamination when an indictor organism is used. Would recommend specifying E. coli or something along those lines. Further, in the introduction, the author talks about the study focusing on Campylobacter seems unnecessary as they never return to this pathogen and the study does not actually look for Campylobacter but an indicator organism, which has unproven status as an indicator in this setting.

**Reply:** We agree with the reviewer’s comment and revised the title as “Quantitative Multi-pathway Assessment of Exposure to *Escherichia coli* for Infants in Rural Ethiopia” and replaced “fecal contamination” with “fecal indicator” or “*E. coli*” throughout the paper. We also removed the contents related to “Campylobacter” from the introduction.

Reviewer #2: Line 45: Instead of saying “basic WASH”, describe what these interventions included so it is clearer what did not work.

**Reply:** We added the details for the “basic WASH” in lines 66-68 as “the investigators concluded that basic WASH interventions (i.e., water chlorination and promotion, latrine improments and promotion, and handwashing stations with soap and hygiene promotion) were not sufficient to reduce pediatric diarrheal disease and improve linear growth”.

Reviewer #3: See Summary Comments

**Summary and General Comments**:

Use this section to provide overall comments, discuss strengths/weaknesses of the study, novelty, significance, general execution and scholarship. You may also include additional comments for the author, including concerns about dual publication, research ethics, or publication ethics. If requesting major revision, please articulate the new experiments that are needed.

Reviewer #1: The paper is an interesting exploration of pathway routes, and the rural landscape is under-explored, making it a worthwhile contribution to the existing body of literature on the topic. As such, the paper should be published but needs significant revision to expand on the methods, the limitations of the study, and to flesh out additional results to improve the robust contributions it makes to literature.

**Reply:** We appreciate the thoughtful and detailed comments by the reviewer. We addressed the feedback carefully and revised the paper to improve the methods, limitations, and results.

Reviewer #2: 1. Some references are inapproriately old (e.g. references from 2012 and 2016 are used in the introduction to introduce the burden of diarrhea).

**Reply:** We updated the outdated references in lines 45-48.

2. Relevant references from other time-activity studies in LMICs are missing and should be included in the intro and discussion section (e.g. Ngure 2013 – geophagy and ingestion of animal feces, Mattioli 2015 – hand-to-mouth contacts vs drinking water, Kwong 2020 - ingestion of fecal bacterial along multiple pathways)

**Reply:** We added relevant references in the paper. We appreciate the careful comments by the reviewer that significantly improved this paper in lines 56-61.

Reviewer #3: The study provides a novel dataset based on observations of child-environment interactions for children in vulnerable age windows of 4-8 months and 11-15 months in rural Ethiopia.

The data are integrated with previously published environmental contamination data using a modeling approach to estimate children's exposures to E. coli through the environment, and highlight child-environment interactions that lead to the greatest risk of disease.

The study is thoughtful and thorough, and well written. The findings are useful for helping to guide decisions around hygiene interventions, and support the need for better access to water, sanitation, and hygiene infrastructure.

**Reply:** We thank the thoughtful comments by the reviewer and carefully address them to improve the paper.

**Major Comments:**
1) The study replicates some earlier work on child-environment observations in other contexts, including the described potential use case of monitoring chemical exposures. The introduction would benefit from an overview of previous work, to highlight that this study -while innovative- is rooted in previous studies using structural observation to understand environmental exposures. How do child behaviours observed here compare to other studies, or are they not comparable due to not standardized methods?

**Reply:** We added some sentences in lines 90-94 to talk about previous exposure assessment works (with five new references) for environmental contaminants and chemicals provided the foundation of this work. As much as we would like to compare the behavior data between this study with our previous studies, a direct comparison is challenging. We only collected behavior sequence data (with behavior and compartment) in Accra, Ghana (2012), Kolkata, India (2018, not published), and Haramaya, Ethiopia (2022). The study design, including subjects, settings, behavior definitions, training, and data collection tool, has evolved over time makes comparing statistics like frequency of certain behavior without careful adjustment become not appropriate. For example, the enumerators are still using paper-based observation, which can only capture time in minutes, in the Ghana study, while we used mobile data collection tools to enable higher resolution (in seconds) in time of observation in India (using ODK) and Ethiopia (using Countee). And we only collected some frequency-based behavior such as touching and mouthing in thie Ethiopia study. We believe this is out of the scope of this paper and have listed it as a limitation in the discussion. We plan to work on another paper to carefully compare the behavior information while acknowledging the differences caused by different study designs.

Table 1. Summarization of structured observation in three study countries

| ***Location*** | **Accra, Ghana** | **Kolkata, India** | **Haramaya, Ethiopia** |
| --- | --- | --- | --- |
| ***Setting*** | Urban | Urban | Rural |
| ***Age*** | 0-5 | 0-5 | 0-1 |
| ***Year*** | 2012 | 2019 | 2021 |
| ***Number of Neighborhoods*** | 4 | 2 | 1 |
| ***Number of Children*** | 154 | 48 | 79 |
| ***Hours of Observation*** | About 500 | 244 | 702 |
| ***Device for Data Collection*** | Paper Based | KoboToolBox | Countee |
| ***Activity Observed*** | Playing/sitting, Sleeping, Handwashing, Bathing, Defecating, Eating | Playing/sitting, Sleeping, Swimming/Splashing, Handwashing, Bathing, Defecating, Eating, Drinking | Awake, Sleeping, Bathing, Drinking |
| ***Compartment Observed*** | Unimproved Ground, Improved Ground, Off Ground, Stagnant Water/Trash Area, Open Drain | Unimproved Ground, Improved Ground, Off Ground, Stagnant Water/Trash Area, Open Drain, Out of View | Carried by Mom, Carried by Others, Down with Barrier, Down Bare Ground |
| ***Frequency Based Behavior*** | NA | NA | Breastfeeding, Mouthing, Pica, Touching |
| ***Social Contact Behavior*** | NA | With Caregiver, With Other Adults, With Other Child, With Animal | NA |

2) The modeling work (Section 2.4) is only discussed in minimal detail, with a reference to the availability of the code. Although this approach is maybe o.k., I think an important topic to discuss is what influence modeling assumptions will have on the major conclusions from the model. For example, are there modeling assumptions that reduce the relative contributions of hand-to-mouth contacts for children? I am very surprised by this finding, given others (see Mattioli et al. ) have shown hand-to-mouth contacts are quite important, and I'm curious what drives this results, modeling framework or observations. More generally: what model assumptions influence outcomes and their interpretation?

**Reply:** We included the details of the exposure model, parameters, and assumptions (2 additional supplementary tables and 1 additional supplementary figure) in the Supplementary Materials. I think our findings are not necessarily different from what Mattioli et al. found in their paper. In their paper, they compare the hand-mouth pathway with stored drinking water, while in our study, we have 10 pathways to compare. In our study, those hand-mouth indirect pathways were more important compared to drinking water or bathing water, but just not the most important (dominant) pathways as drinking breastmilk, eating food, or pica soil.

**Minor Comments:**
1) Abstract should be readily understood, so I suggest describing Countee as no one will be familiar with this, and also replacing Timepoint 1 and Timepoint 2 with the age ranges of the children (4-8 mo; 11-15 mo, if I understand correctly). Generally replace Timepoint 1 and 2 with age ranges throughout whole manuscript to improve readability.

**Reply:** We revised the sentence and described the Countee app as “a data collection system for behavioral data”. We agree that linking Timepoint with the actual age range could improve the readability of some results and interpretations. However, this was a study designed to understand the difference between exposures of infants at two developmental phases (1–3 months and 6–9 months) driven by their behavior changes. As we described in the limitation, the concurrent impact of the COVID-19 pandemic and geopolitical tension within the country delayed our plan. While replacing Timepoints 1 and 2 with the actual age range does improve the readability, it sacrifices the original idea of the study design. For this reason, we believe using Timepoint 1 and Timepoint 2 is the better choice to keep the original “flavor” of this study. Meanwhile, the contamination level of the environmental samples (e.g., drinking water) is not impacted by the age of the infant. Therefore, in such a case, reporting the timepoint instead of the age range is more appropriate. To improve the readability, we added the actual age ranges after the Timepoint in the results and discussion sections wherever it was appropriate.

2) 32-34 - these are old numbers, are there updated numbers available?

**Reply:** We updated the numbers and references in lines 45-48.

3) 46-51 - consider reframing, the argument here is not clear. For example, you could establish that a mechanistic explanation of the conclusions of the RCTs would improve understanding; and fecal contamination along exposure ways may help to establish or validate a mechanistic explanation for why interventions do/do not reduce exposures and subsequent diarrhea.

**Reply:** We reframed those sentences in lines 64-74.

4) 93 - describe CAGED trial if referring to it is important; audience will not be familiar with it.

**Reply:** Agreed and revised in lines 115-119.

5) 102-109: a comment would be helpful to describe the degree to which these specific categories of activities are location specific, or sufficiently generalizable.

**Reply:** The categories of behaviors and compartments are sufficiently generalizable among the same age group. The subcategories can be location-specific. For example, infants eat solid food, which can differ between countries. We added a sentence in lines 159-161 to describe this: “The categories of behavior, compartment, and location defined in this study are sufficiently generalizable among the same age group.”
6) 166 - child's hands?

**Reply:** Yes, we revised it.
7) Can you use and describe statistical tests to compare results between the two time points (Table 1 results), as differences are discussed in lines 196-201.

**Reply:** We revised in lines 221-223 in the methods section: “In addition, we conducted Wilcoxon signed-rank tests to compare the percent of time spent for sepecific duration-based activity, compartment, and location, as well as the rate of specific frequency-based activity between paired observations at Timepoint 1 and Timepoint 2.” We also added the p-values in the text, Table 1, and Table 2.

8) 228 - give stats test and metrics when stating something is statistically different.

**Reply:** Revised in lines 221-224 in the methods section. We added the statistical test information in the caption of Table 3 and we added all the p-values in the text.

9) 249 - what assumptions in the modeling framework influence this? Why does this deviate from Mattioli et al. findings? DOI: <https://doi.org/10.1021/es505555f>. See major comment.

**Reply:** We explained this in our reply to the major comment.

10) 262 - how is this well calibrated?

**Reply:** In the previous study (Wang et al., 2018: <https://doi.org/10.1111/risa.13146>), we modeled the transfer coefficient (attachment and detachment) during repeat hand contact (subsection 2.3.3). We also compared the hand contamination predicted by the model with the observed numbers of microbes in the hand rinse samples from the study neighborhoods (Figure 6) as part of the validation. We agree that “well-calibrated” could be an overstatement so we removed it.

11) 274-298 - some assertions without references made, suggest supporting statements in this section with evidence.

**Reply:** Thanks for the suggestions. We added four references to support the statements related to the infant’s behavior that we made.

12) 284-288 - it is well established that diarrheal disease risks increase for older children compared to younger children; suggest connecting your findings to those studies.

**Reply:** Agreed. In lines 367-369, we added: “Studies (Mulatya and Ochieng, 2020; Kombat et al., 2024) reported that children aged 6-23 months were found to be much more likely to experience diarrhea compared to those under 6 months, which could be driven by their changing behavior. ”

13) 311-315 -authors neglect to describe their results in the context of other studies on structured observations of children. Suggesting linking results to other published results. See major comments.

**Reply:** Based on our replies to the major comments, we can not do a direct comparison with previous studies. We added this as a limitation of this study in lines 456-458.

14) 344 - multiple studies looking at children's exposures to environments have been done to characterize chemical exposures (see for example U.S. EPA Children's Exposure Factors Handbook). Suggest authors better describe this in the introduction (see Major Comment)

**Reply:** We added five new references, including the EPA Exposure Factors Handbook, and described this in the introduction.
15) 365 - new paragraph?

**Reply:** It is a new paragraph (LaTeX did not add space between paragraphs). This will be naturally resolved by the publication team when fits into their template.
16) 360 - Timepoints are clearly linked to age ranges here; suggest using age ranges, not time points (see minor comment 1).

**Reply:** Please see the reply above for the minor comment 1.
17) Discussion - comment on the degree to which these findings for children are specific to this location, or are generalizable to other settings and scenarios. Are we supposed to conduct similar studies everywhere we want to decide WASH interventions, or can these findings be applied to new areas?

**Reply:** We added a sentence in lines 472-476: “While human behavior and environment are dynamic and location specific, which means we need to collect new data for each assessment, the overall multi-pathway exposure assessment design (e.g., categories of behavior, environmental pathways) can be very similar across settings and countries, making the data collection and modeling framework generalizable.”

PLOS authors have the option to publish the peer review history of their article ([what does this mean?](https://nam11.safelinks.protection.outlook.com/?url=https%3A%2F%2Ftrack.editorialmanager.com%2FCL0%2Fhttps%3A%252F%252Fjournals.plos.org%252Fplosntds%252Fs%252Feditorial-and-peer-review-process%2523loc-peer-review-history%2F1%2F010f019503df0a44-9f9119a2-2688-4668-863d-a5fb57c99dc7-000000%2FmFBGesQORZH11L54u3nrdpZHv1d9TpW5BHN7goYgqSE%3D198&data=05%7C02%7Cyuke.wang%40emory.edu%7C3ee9af123157438e220408dd4cdd3095%7Ce004fb9cb0a4424fbcd0322606d5df38%7C0%7C0%7C638751235083530527%7CUnknown%7CTWFpbGZsb3d8eyJFbXB0eU1hcGkiOnRydWUsIlYiOiIwLjAuMDAwMCIsIlAiOiJXaW4zMiIsIkFOIjoiTWFpbCIsIldUIjoyfQ%3D%3D%7C0%7C%7C%7C&sdata=I6ZbNJwfpeE2U61vkMtBuNKO%2B0Ls%2ByG4Ip9sv4lHH84%3D&reserved=0)). If published, this will include your full peer review and any attached files.

If you choose “no”, your identity will remain anonymous but your review may still be made public.

**Do you want your identity to be public for this peer review?** For information about this choice, including consent withdrawal, please see our [Privacy Policy](https://nam11.safelinks.protection.outlook.com/?url=https%3A%2F%2Ftrack.editorialmanager.com%2FCL0%2Fhttps%3A%252F%252Fwww.plos.org%252Fprivacy-policy%2F1%2F010f019503df0a44-9f9119a2-2688-4668-863d-a5fb57c99dc7-000000%2FIwiFLITefpczYmoNkuTwJr7KHK31YZFMvZG8e8s0V_E%3D198&data=05%7C02%7Cyuke.wang%40emory.edu%7C3ee9af123157438e220408dd4cdd3095%7Ce004fb9cb0a4424fbcd0322606d5df38%7C0%7C0%7C638751235083566754%7CUnknown%7CTWFpbGZsb3d8eyJFbXB0eU1hcGkiOnRydWUsIlYiOiIwLjAuMDAwMCIsIlAiOiJXaW4zMiIsIkFOIjoiTWFpbCIsIldUIjoyfQ%3D%3D%7C0%7C%7C%7C&sdata=0MvwJKsqxiHJxvYNdLp7sn5qWaY3bKFp4W9i3y9otbc%3D&reserved=0).

Reviewer #1: No

Reviewer #2: No

Reviewer #3: No

[NOTE: If reviewer comments were submitted as an attachment file, they will be attached to this email and accessible via the submission site. Please log into your account, locate the manuscript record, and check for the action link "View Attachments". If this link does not appear, there are no attachment files.]

**Figure resubmission:**

While revising your submission, please upload your figure files to the Preflight Analysis and Conversion Engine (PACE) digital diagnostic tool, <https://pacev2.apexcovantage.com/>. PACE helps ensure that figures meet PLOS requirements. To use PACE, you must first register as a user. Registration is free. Then, login and navigate to the UPLOAD tab, where you will find detailed instructions on how to use the tool. If you encounter any issues or have any questions when using PACE, please email PLOS at figures@plos.org. Please note that Supporting Information files do not need this step. If there are other versions of figure files still present in your submission file inventory at resubmission, please replace them with the PACE-processed versions.

**Reproducibility:**

To enhance the reproducibility of your results, we recommend that authors of applicable studies deposit laboratory protocols in protocols.io, where a protocol can be assigned its own identifier (DOI) such that it can be cited independently in the future. Additionally, PLOS ONE offers an option to publish peer-reviewed clinical study protocols. Read more information on sharing protocols at <https://plos.org/protocols?utm_medium=editorial-email&utm_source=authorletters&utm_campaign=protocols>
